# Influence of the three-body abrasion kinematics on the surface characteristics of an SLS-fabricated tool during machining of ceramics

**DOI:** 10.1038/s41598-025-02692-7

**Published:** 2025-06-05

**Authors:** Sisay Workineh Agebo, Dawid Zieliński, Mariusz Deja

**Affiliations:** 1 Doctoral School, Gdańsk University of Technology, Gabriela Narutowicza Street 11/12, 80 - 233 Gdansk, Poland; 2 Department of Manufacturing and Production Engineering, Faculty of Mechanical Engineering and Ship Technology, Institute of Machine and Materials Technology, Gdańsk University of Technology, Gabriela Narutowicza Street 11/12, 80 - 233 Gdansk, Poland

**Keywords:** Three-body abrasion, Selective laser sintering, Tool wear, Ceramic materials, Engineering, Mechanical engineering

## Abstract

Experimental and modeling assessments have been conducted on the surface characteristics of SLS-printed polyamide lapping tools, considering the influence of three-body abrasion kinematics during machining of Al_2_O_3_ ceramic materials. Based on the radial axis profile and topography of segments, surface characteristics were assessed using 3D optical profilometers and minimum zone (MZ) techniques. Machining with counter-rotational kinematics exhibited larger surface shape error, resulting in an average maximum height (Wt) of 163.48 µm and pinpointing intense wear at a tool radius of 95 mm. Conversely, the highest wear with co-rotational kinematics resulted at a tool radius of 85 mm. Ceramic materials were improved by 64.74%, enhancing the initial spatial roughness Sa 1.73 µm to Sa 0.61 µm and resulting in near-zero skewness (Ssk) of surface height distribution with co-rotational kinematics. The three-body abrasion with counter-rotational kinematics resulted in 17.14% higher material removal than co-rotational kinematics. The tool-workpiece contact has been modeled considering the influence of the workpiece’s velocity and tangential acceleration along the active surface, and the findings confirmed experimental observations.

## Introduction

Owing to improved chemical and physical properties, advanced ceramic materials such as SiC, Al_2_O_3_, Si_3_N_4_, and ZrO_2_ are mostly suitable in the application area requiring resistance to wear and high temperature. Nowadays, ceramic materials are more preferred and popular in biomedicine, tool industries, and aerospace applications due to their hardness, strength, resistance to wear, poor thermal conductivity, and biocompatibility^1–5^. Additionally, ceramic material, specifically technical Al_2_O_3_ is highly in demand in areas requiring stability with chemical, thermal, and corrosion, also in the need of piezoelectricity and light transmission. However, due to its superior and difficult-to-cut properties, machining of advanced ceramic materials is challenging^6^.

In precision industries, abrasive machining is typically utilized to acquire close tolerance and improved surface finish on the workpiece. Lapping is a basic abrasive machining technique in which flattening of the workpiece surface can be achieved using a single plate—one-sided lapping^7–9^ or two plates—double-sided lapping^10–12^. Based on the abrasive particle usage to obtain global flatness on the workpiece, the lapping process can also be classified as free abrasive and fixed abrasive lapping processes^13,14^. During the lapping process, the removal of materials occurs due to three-body abrasion having free movement of abrasive grains for the free abrasive lapping process and by two-body interaction for the fixed abrasive lapping process due to immobilized fixed abrasive grains in the lapping plate^15,16^. In industrial applications, lapping plates made of metals are mostly preferable due to their easier fabrication process and the resulting better material removal rate. Conventionally, lapping processes were mostly performed using plates made from cast iron (Fe) and copper (Cu)^17–19^. Whereas, due to the advancement of modern fabrication processes nowadays, additive manufacturing was found in a prominent direction for the production of abrasive tools^20^. Additive manufacturing methods such as stereolithography (SLA)^21^, selective laser sintering (SLS)^22^, and ultraviolet (UV)-bonding^23^ were widely used in the fabrication of lapping tools. Compared to lapping plates constructed of slurry-based cast iron, resin-bonded diamond abrasive grains used in ceramic material machining resulted in a lower average roughness parameter Ra^24^ and a greater material removal rate^25^. The lapping tools fabricated from polyamide powder PA2200 using the selective laser sintering (SLS) 3D-printing technique were found to be significant in improving the surface and removing materials from the difficult-to-cut Al_2_O_3_ technical ceramics with the occurrence of a lower amount of wear in the tool surface even after long-lasting machining^22,26^.

Despite advances in the fabrication of abrasive tools, the lapping process is still complex, with multiple controlled and uncontrolled input factors^27,28^. The kinematics configuration^29,30^, processing parameters^31^, arrangement, type, and sizes of the abrasive grain^32,33^, workpiece state of stress^34^, and lapping fluid type and properties^35–37^ can significantly influence the performance of lapping process. The three-body abrasion during the lapping process is also considered one of the significantly influencing factors on the tool’s flatness error, simultaneously causing the active surface of the tool to gradually change its shape, leading to experiencing concavity, convexity, or axial runout profiles, resulting in poor dimensional accuracy of the workpiece^38,39^. Pan et al.^40^ assessed the surface shapes of planetary wheel in the mechanism of concavity and convexity formation by considering the effect of workpiece eccentricity and rotational speed. The wheel rotational speed and eccentricity take over the tool’s concavity and convexity, increasing its flatness. However, the trajectory also found significantly affecting the wear on the wheel surface. Lai et al.^41^ investigated the wear characteristics of double-sided lapping tools considering the trajectory distribution when machining sapphire slices. The modeling and experimental results revealed that trajectory distribution had a considerable impact on the tool profile along the radial axis. Uhlmann et al.^42,43^ used trajectory analysis to examine the wear characteristics of lapping wheels at different rotational speeds. When compared to high rotational speeds, lesser rotational speeds resulted in much higher wear. In reference^44^, the wear of the lapping tool was also assessed considering the influence of the rotational speed and acceleration of the lapping process. An empirical formula was developed for wear considering the trajectory distribution, workpiece motion velocity, and acceleration. The model was further validated with the results of experimental investigations.

Moreover, many studies also explored to determine the lapping process uniformity by considering an additional movement of the guiding ring along the lapping plate. Piotrowski^45^ used a computer model and data-driven technique to simulate abrasive grain trajectories for the single-sided lapping process while the lapping plate and conditioning ring rotate in the same and opposite directions. The study considered tool wear uniformity and lapping parameters with the position of the conditioning rings and the rotational speed of the lapping plate and conditioning rings. The center position of the conditioning ring produced the maximum uniformity of lapping plate wear, as did the ratio of rotating speeds of the ring and the lapping plate, which generated pericycloidal trajectories. Additionally, results from the unconventional lapping system proved that the introduction of additional and controlled movement of the guiding ring can change the density of grain trajectories, allowing the occurrence of a more even wear on the lapping plate^17^. Lai et al. ^46^ assessed the lapping wheel’s wear while taking the carrier eccentricity into account. The bigger workpiece size and placing it farther from the carrier center at a specific ratio of rotating speed has a substantial impact on the wheel’s lifetime and wear uniformity. Thus, the proper settings of parameters and the lapping kinematics setup can extend the tool life. The authors of the research^47^ assessed lapping uniformity using the workpiece swinging mode of motion and considering the relative motion between the workpiece and fixed abrasive pad. The findings revealed that the extra swinging prevented the periodic distribution of material removal, which had a substantial effect on lapping efficiency and uniformity.

Overall, the literature analysis indicates a lack of research in evaluating the influence of three-body abrasion kinematics on the surface characteristics of lapping tools, particularly for additively manufactured tools. Since of the increasing improvement of tools and fabrication processes, it is highly advised to evaluate the newly fabricated tool’s performance because it gives a comprehensive understanding of the wear characteristics and insight to enhance tool design for better technical impacts and performance. The aim of this study is to assess the influence of three-body abrasion kinematics on the surface characteristics of SLS-printed polyamide lapping tools, considering co- and counter-rotational kinematics while machining the ceramic materials Al_2_O_3_. Two polyamide lapping tools, each with eight segments, were utilized during single-sided free abrasive lapping, and the surface characteristics along the radial axis of the segments were assessed using a non-contact type 3D optical profilometer based on minimum zone (MZ) techniques, surface topography, and the waviness maximum height (Wt) surface parameter. Furthermore, modeling has been performed to understand contact intensity within the kinematics configuration, taking into account the tool-workpiece contact, workpiece velocity, and tangential acceleration along the active region of the lapping tool. The assessment revealed the highest wear location for kinematics setups pinpointing along the radial length of the segments. The experimental and modeling results confirm that kinematics configurations had different trajectory distributions, resulting in two distinct intense wear locations on the tool radial length.

## Materials and methods

### Experimental setup and investigations

**Fig. 1:**
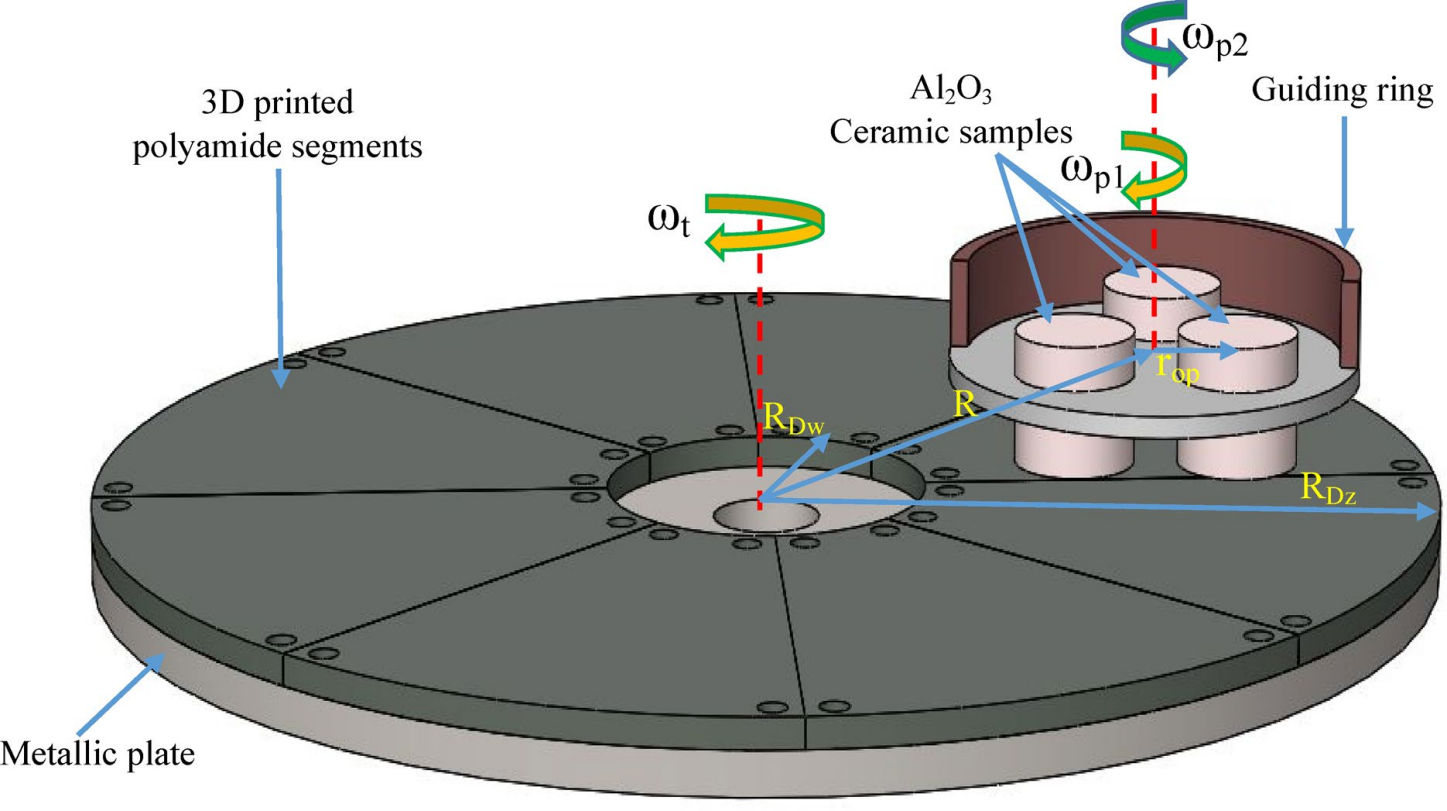
Schematics of the experimental and simulation setup with eight polyamide lapping segments fixed on a metallic plate of the lapping machine (Details in Table 3).

The influence of lapping kinematics on the surface characteristics of an additively fabricated polyamide PA12 tools have been investigated during flat lapping of Al_2_O_3_ ceramic materials. A two sets of lapping tools, each consisting of eight segments, were fabricated using Lisa X SLS printing system following the parameters listed in Table 1. An industrial PA12 polyamide powder material from Sinterit having a property listed in Table 2 was utilized to build segments with layer-by-layer sintering process. As illustrated in Fig. 1, experimentations were conducted in prototype lapping machine configured with two independent controllable steeper motors for the lapping plate and guiding ring, allowing to machine Al_2_O_3_ ceramic materials with co-rotational and counter-rotational kinematics configurations.

**Table 1:** SLS printing parameters used for the fabrication of polyamide lapping tools.

3D printing technology and system	Powder material	
Selective laser sintering: Lisa X	PA12 industrial polyamide	

3D printing parameters	Values	Unit
Single layer thickness	125	µm
Power of IR fiber coupled diode laser	30	W
Printing speed	0.212	mm/min
Laser wavelength	976 ± 3	nm
Laser spot size	650	µm
Printing orientation	Vertical	
Post-printing		
Sandblasting and cleaning with a compressed air

**Table 2:** Selected mechanical and general properties of polyamide PA12 industrial^48^.

Properties	Value		Units
	(X direction)	(Y direction)	
Mechanical properties			
Tensile modulus (Young)	2001	1983	MPa
Tensile strength	47.61	48.66	MPa
Flexural strength	62.31	57.55	MPa
Impact strength (Charpy—unnotched)	15.23	22.92	kJ/m^2^
Shore D hardness	75		–
General properties			
Bulk density	505		kg/m^3^
Printout density	0.99		g/cm^3^
Mean particle size D50	62		µm

An SLS-printed lapping tools with a specifications and conditions given in Table 3, were used during experimental investigations and simulation. In each experimental investigations three Al_2_O_3_ ceramic material samples with the given surface and physical properties were considered for the evaluation of technological effects in terms of surface characteristics.

**Table 3:** Physical and surface condition of the lapping process and Al_2_O_3_ ceramic materials.

Specification	Symbol	Value	Units
Lapping plate			
Tool outer radius	R_Dz_	190	mm
Tool inner radius	R_Dw_	45	mm
Average initial surface roughness	*Sa*	14	µm
Guiding ring configuration			
Distance to center of guiding ring from the center of lapping tool	*R*	115	mm
Distance to center of workpiece from the center of guiding ring	*r*_*OP*_	35	mm
Al_2_O_3_ ceramic materials			
Vickers hardness	HV10	1100 MPa	
Sample shape and diameter	*d*_*w*_	cylindrical with diameter 34 mm	
Initial height	*h*_*w*0_	30 mm	
Average initial surface roughness	*Sa*	1.73 µm	

The experimental tests conducted with kinematics configurations of co- and counter-rotational lapping, with a set of two lapping plates. Table 4 gives the lapping parameters and abrasive suspensions used in the experimental investigations based on^22,26^. To account the effect of kinematics configurations and input parameters on the wear characteristics of the lapping tool and the technological effects, experiments divided in both kinematics configurations into a three series of tests, in which each series of machining conducted for 120 min.

**Table 4:** Experimental conditions during lapping Al_2_O_3_ ceramic materials using SLS-fabricated tool.

Parameters	Symbol	Value	Unit
Lapping parameters			
Unit pressure	*p*	12	kPa
Speed of the lapping plate	*n*_*t*_	120	min^−1^
Speed of the guiding ring	*n*_*w*_	60	min^−1^
Duration of lapping for single series	*t*_*t*_	120	min
Total lapping duration for each kinematics configuration	*Tt*	360	min
Abrasive suspension			
Abrasive suspension type and its volume during each series of lapping run		Abrasive paste SD 1/0 (4 ml)	
		Loose diamond abrasive grains D107 (2.5 ml)	
Lubricant type and PH		Machining oil, 5.60	
Lubricant flow rate	${\dot{\text{Q}}}_{\text{lub}}$	0.1 ml/min	

### Material removal mechanism of single-sided free abrasive flat lapping

The effectiveness of any machining operation can be evaluated by the resulting technological effects. Lapping operation is mostly used to obtain exceptional surface quality with lesser amount of material removal, and its effectiveness can be assessed either in terms of the amount of materials removed from the workpiece or based on the obtained quality score, such as surface roughness and surface flatness. The material removal mechanism of the lapping process was known for its complexity and can be influenced by multiple factors.

**Fig. 2:**
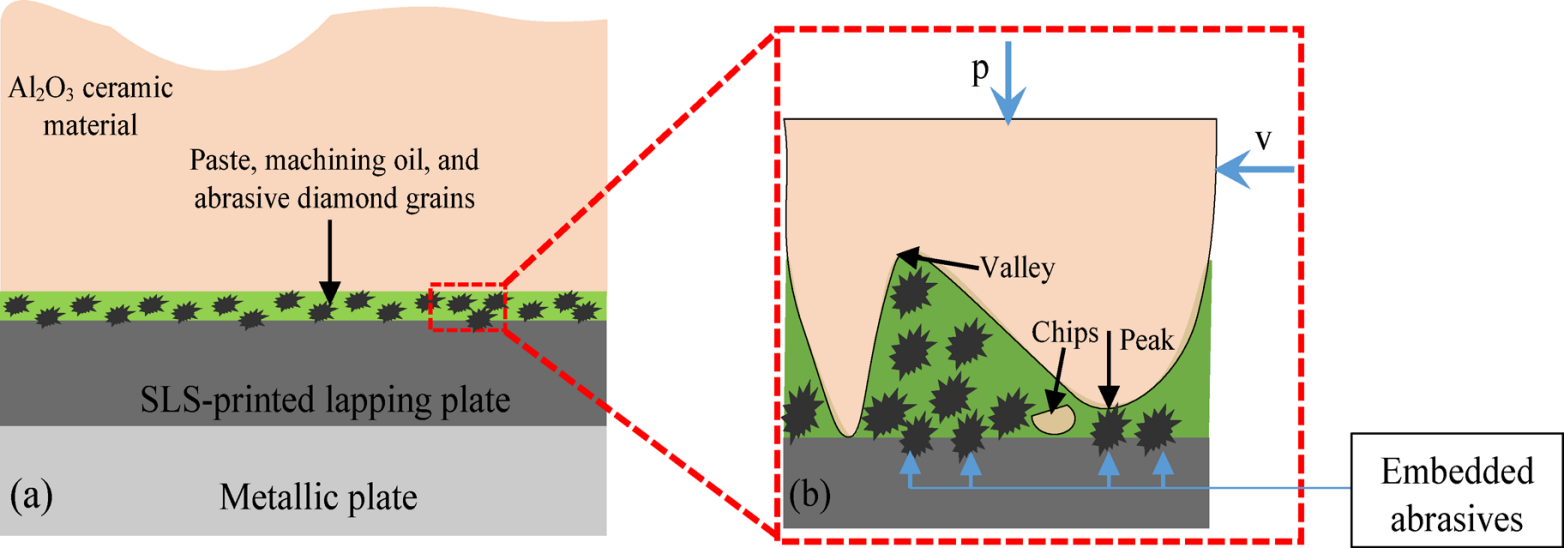
Schematic of (**a**) material removal mechanism of single-sided free abrasive flat lapping, (**b**) detailed view.

In this study, a single sided free abrasive lapping of Al_2_O_3_ ceramic materials was carried out, and the removal of material occurs due to the interaction of three bodies, such as the lapping plate, workpiece, and the diamond abrasive particle. During the two kinematics configurations of each series of experimental tests, the abrasive suspensions (abrasive paste SD 1/0 and loose diamond abrasive grains D107) with amounts given in Table 4 were applied to the surface of the lapping plate before machining the ceramic materials Al_2_O_3_. The unit pressure applied into the workpiece top surface and the relative motions between the lapping plate and the guiding ring result in the abrasive particles being embedded^26^ in the active surfaces of the lapping plate and trapped between the plate and workpiece, acting as a cutting tool and removing a material in the form of chips—Fig. 2. Generally, various factors can influence the material removal mechanisms, including the abrasive size and hardness, samples properties, lapping speed, unit pressure, and lubricants. The presence of lubricants will impact the material removal mechanism through reducing friction and fostering plastic deformation. In this study, material removals during co- and counter-rotational kinematics configurations were measured after consecutive 20 min of lapping using a digital mass balance with resolutions of 0.001 µm and 0.001 g, respectively, considering the change in weights from the samples, which has a significant impact on dimensional accuracy, surface finish, and overall quality scores of the lapping process.

### Measurement procedures

#### Techniques used to acquire a radial axis profile and topography of the tool

**Fig. 3:**
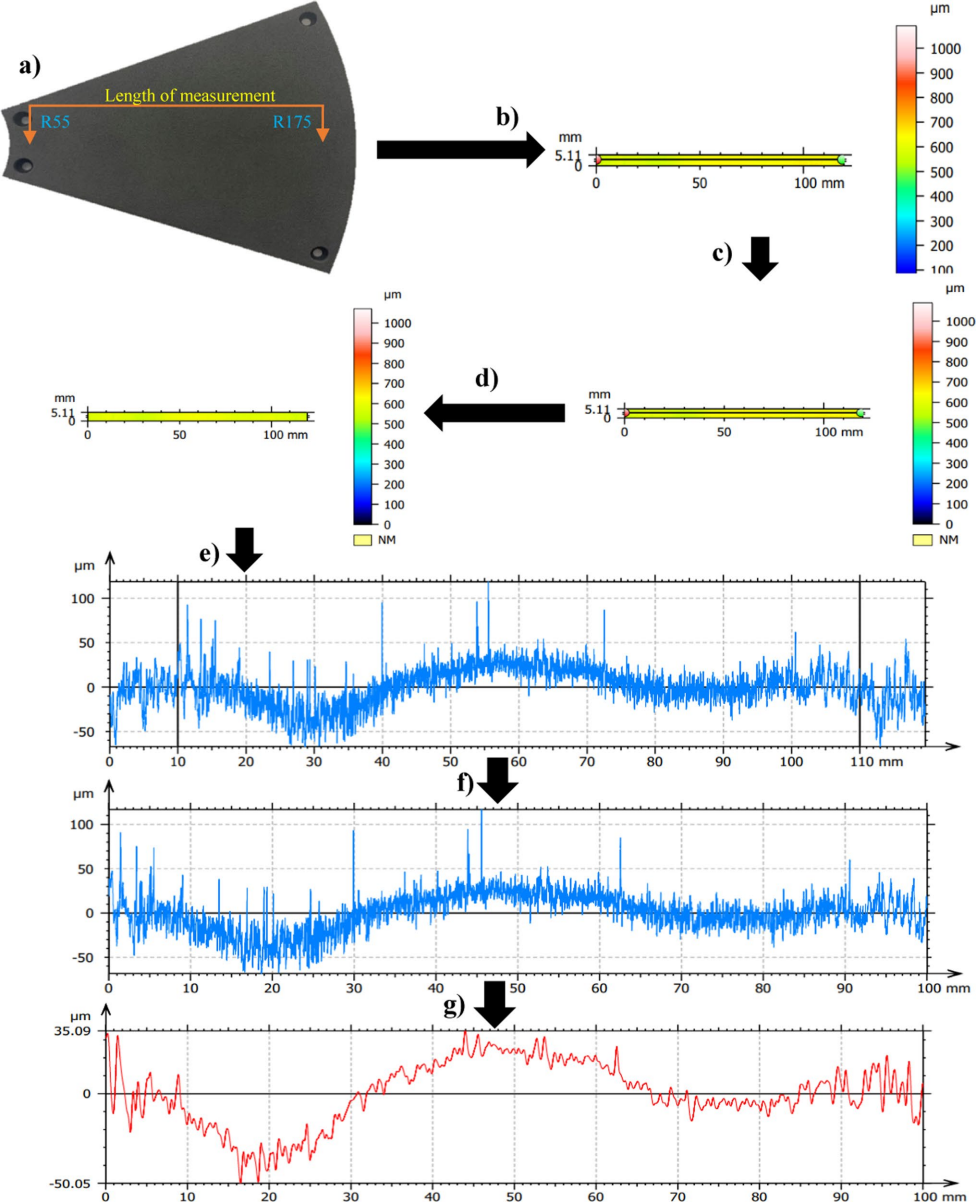
Exemplary measurement and analysis procedure of the radial axis tool profile on the lapping segment #4 after series no. 3 (360 min) of co-rotational lapping: (**a**) lapping tool with its measuring radial axis, (**b**) topography channel of radial axis, (**c**) filled-in non-measured points, (**d**) levelling (MZ), (**e**) radial profile of the lapping segment, (**f**) extracted profile of contact area, (**g**) waviness profile along the contact area.

The influence of kinematics configurations on the wear characteristics of SLS-printed polyamide lapping tools was assessed by measuring the radial axis of the lapping segments after each series of tests utilizing the methods developed in^49^ as described and illustrated in Fig. 3. Continuous measurements were performed using Sensofar S neox 3D optical profilometer along the radial length of the segments to acquire the surface topography based on the contact and non-contact radial lengths. The measurements were performed by utilizing the confocal 3D expert measurement method and a 5X magnification scale, where the topography of lapping segments was obtained with ~ 98% measured points. The surface topographies after the measurement were further analyzed using SensoMAP Premium 9.1 software. On the topography channel of the measurement, fill-non-measured points were applied to get rid of the measurement errors and refine the measurements. The minimum zone (MZ) technique is used to level the measured topography, which removes residual slopes from the measured radial axis tool profiles.

The measured radial axis tool profile was made for the analysis to visualize the contact and non-contact regions. However, contact length radial axis profiles were considered to assess the wear characteristics of the lapping segments with the radial length of 100 mm. The surface deviations were evaluated using the waviness profile and the parameter maximum height (Wt), where the waviness profile is extracted using a Gaussian filter with cut-off 0.80 mm.

**Fig. 4:**
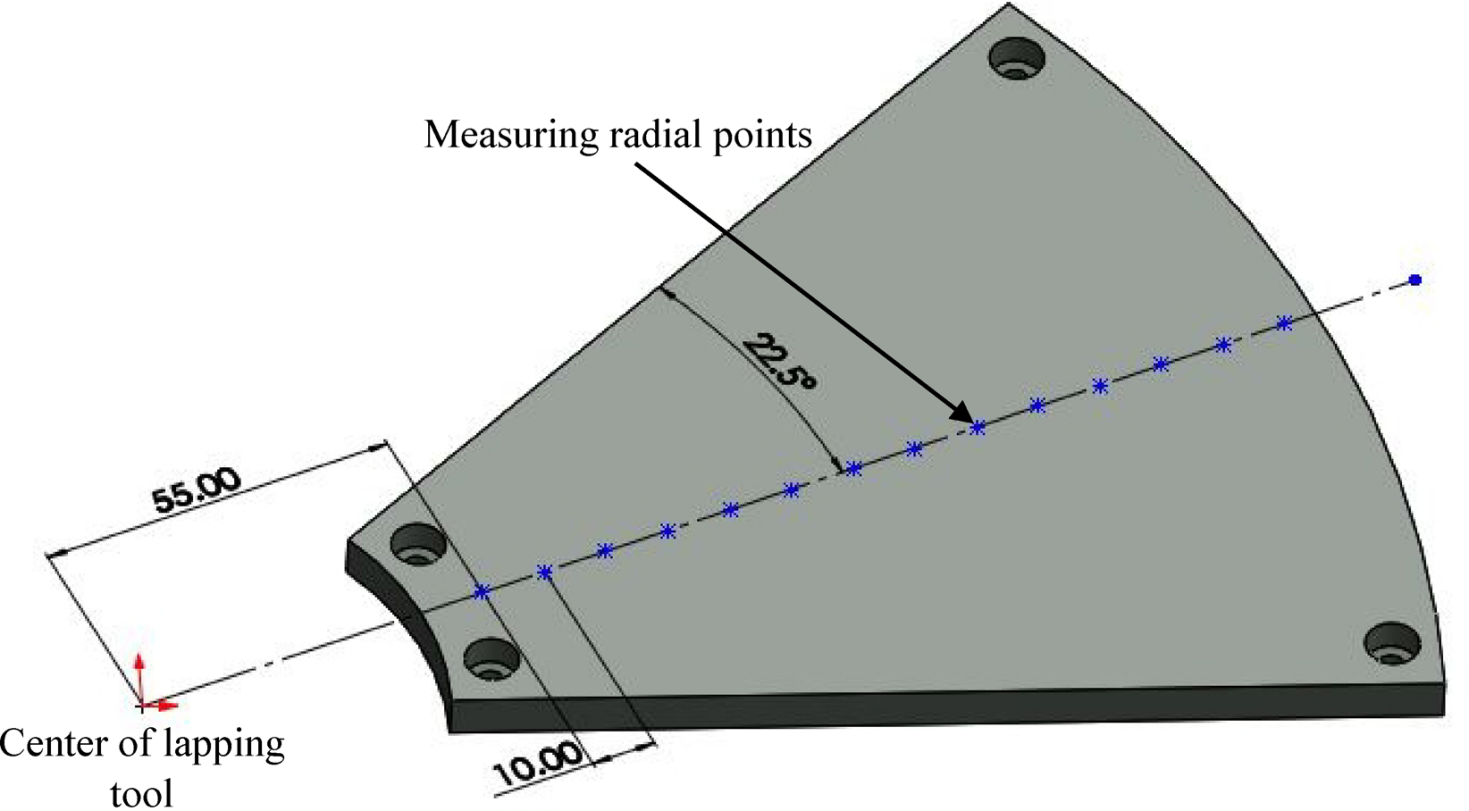
Schematics of lapping tool segment topography with the measuring radial points.

The surface topography of lapping segments was taken into account to evaluate the effect of contacts during machining, which is used to evaluate and determine the highest and lowest contact locations based on the measured results of 3D surface parameter spatial roughness. Measurements were performed on both kinematics configurations after the third series of tests based on standard ISO 25,178 and using confocal 3D expert methods with a 20X magnification scale. A single point topography of the segments radial point was collected from points starting at radius 55 mm by assuming a space with 10 mm until the tool radial point reached 185 mm, based on the polar grid strategy as illustrated in the procedure in Fig. 4.

#### Techniques used for the assessment of ceramic materials surface characteristics

To account for the technological effects of 3D-printed polyamide lapping segments during co- and counter-rotational lapping, measurements on the ceramic samples were conducted after consecutive 20 min of machining. Measurements on the machined flat surface of ceramic samples were carried out in three locations using a Sensofar S neox 3D optical profilometer based on measurement standards of ISO 25,178 and the confocal 3D expert method with a 20X magnification scale. The obtained 3D topography with the surface height parameters was then used for the assessment of Al_2_O_3_ ceramic materials surface characteristics.

## Modeling of tool-workpiece contact within kinematics configurations

The kinematics of three-body abrasion during co- and counter-rotational lapping often has different trajectories along the active surfaces of the tool while leading to different tool shapes and profiles, deteriorating the flatness of the workpiece. The prediction and determination of tool wear in the lapping process require the assessment of the interactions between the lapping tool and workpiece, which are created during co- and counter-rotational lapping. The geometrical relations based on the workpiece diameter has significant influences on the contact intensity between the workpiece and lapping tool. Based on the distribution of velocity and acceleration of the kinematics, contact intensity of the workpiece-tool, and normalized contact density along the lapping surface, a comprehensive tool wear model has been developed using Matlab® software. This numerical assessment was performed by dividing the active surfaces of lapping segments into numbers of individual rings and considering the length of the trajectory traveled by the middle point of the workpiece elementary area along the area of individual rings. However, calculations are conducted by taking into account the difference in time between the entry and exit from the ring, as indicated in^22,44^.

**Fig. 5:**
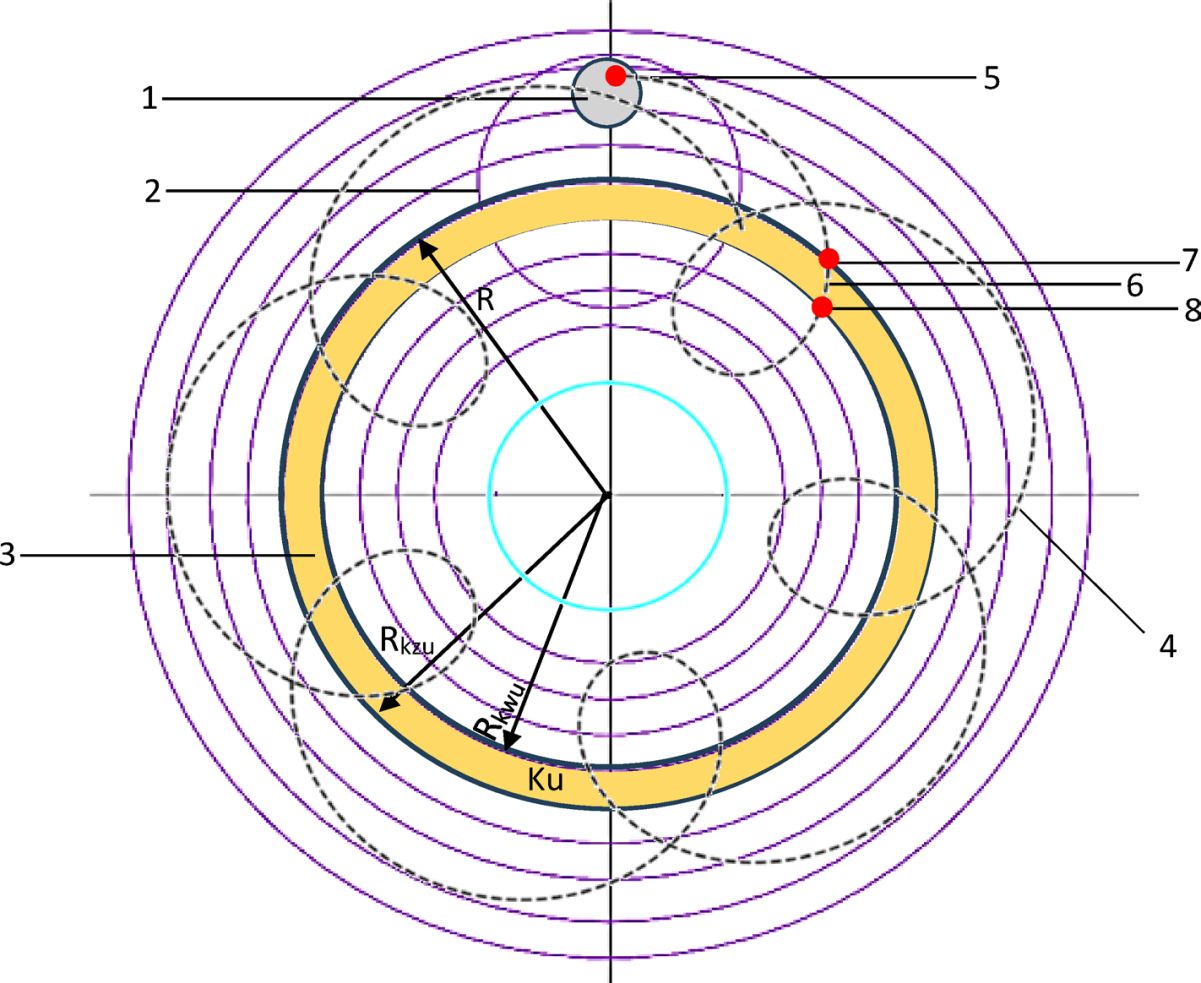
Schematics of lapping trajectory showing 1) workpiece, 2) guiding ring, 3) ring number u, 4) single point (P_1i_) trajectory, 5) workpiece single point (P_1i_), 6) travel length of point (P_1i_) along ring u (∆siu), 7) entry of point P_1i_ into ring Ku at time t_piu_, and 8) exit of point P_1i_ from ring Ku at time t_kiu_.

The modeling has been conducted based on the workpiece single-point trajectory traveling along a specific tool ring area as described in Fig. 5 and calculations performed using the Eqs. (1–11). The length of the trajectory ∆s_iu_ by the workpiece point P_1i_ of an elementary area A_1i_ along the specific ring area Ku during the entry and exit periods at time t was determined using Eq. (1).

1 $$\Delta {s}_{iu}=\underset{{t}_{piu}}{\overset{{t}_{kiu}}{\int }}{v}_{{p}_{1i}}\left(t\right)dt$$

where: v_p1i_ – Velocity of the workpiece single point.

The tool-workpiece contact density for all n_4_ elementary areas A_1_ is calculated using Eq. (2) as a function of lap radius.

2 $${d}_{\text{g}}\left({R}_{D}\right)={\underset{{m}_{1}\to \infty }{\text{lim}}\left[{A}_{1} \cdot \underset{{n}_{4}\to \infty }{\text{lim}}\sum_{i=1}^{{n}_{4}}\Delta {s}_{iu}\right]}_{u=1,\dots ,{m}_{1}}$$

where: A_1_- Workpiece elementary area, n_4_ – Total number of the elementary areas on the workpiece.

The workpiece action rises around the tool center based on the specified contact density because of the rings area. Calculations have been made to account for the variations in the tool ring areas for corrected contact density d_gp_:

3 $${d}_{\text{g}p}\left({R}_{D}\right)={\underset{{m}_{1}\to \infty }{\text{lim}}\left[\frac{{A}_{1}}{2\pi {R}_{ku}\Delta w} \cdot \underset{{n}_{4}\to \infty }{\text{lim}}\sum_{i=1}^{{n}_{4}}\Delta {s}_{iu}\right]}_{u=1,\dots ,{m}_{1}}$$

where: ∆w = R_kzu_ – R_kwu:—_width of the ring k_u_.

The distribution of contact density of all elementary areas was normalized using Eq. 4 based on one cycle time trajectory, with the density R_D_ = R:

4 $${\text{d'}} {\text{g}}\left( {R_{D} } \right) = \left. {\frac{{d_{{{\text{g}}p}} \left( {R_{D} } \right)}}{{d_{{{\text{g}}p}} \left( R \right)}}} \right)$$

To account for the significant effect of velocity and tangent acceleration on the wear rate of the tool, calculations have been made based on their distribution along the active surface of the tools. The average velocity dv was determined using Eq. (6):

5 $${d}_{\text{v}}\left({R}_{D}\right)={\underset{{m}_{1}\to \infty }{\text{lim}}\left[\overline{v }(u)\right]}_{u=1,\dots ,{m}_{1}}$$

6 $${d}_{\text{v}}\left({R}_{D}\right)={\underset{{m}_{1}\to \infty }{\text{lim}}\left[\frac{\sum_{i=1}^{{n}_{5u}}\frac{\Delta {s}_{iu}}{({t}_{kiu}-{t}_{piu})}}{{n}_{5u}}\right]}_{u=1,\dots ,{m}_{1}}$$

where: n5u: the number of elementary areas in contact with the ring ku.

Following the determination of velocity distribution, tangential acceleration was also determined based on Eq. (8):

7 $${d}_{\text{a}}\left({R}_{D}\right)={\underset{{m}_{1}\to \infty }{\text{lim}}\left[\left|\overline{{a }_{t}}(u)\right|\right]}_{u=1,\dots ,{m}_{1}}$$

8 $${d}_{\text{a}}\left({R}_{D}\right)={\underset{{m}_{1}\to \infty }{\text{lim}}\left[\frac{\sum_{i=1}^{{n}_{5u}}\frac{\left|\Delta {v}_{p1iu}\right|}{({t}_{kiu}-{t}_{piu})}}{{n}_{5u}}\right]}_{u=1,\dots ,{m}_{1}}$$

where:

9 $$\Delta {v}_{p1iu}=\left({v}_{p1iu}\left({t}_{kiu}\right)- {v}_{p1iu}\left({t}_{piu}\right)\right)$$

Also ${v}_{p1iu}\left({t}_{kiu}\right), {v}_{p1iu}\left({t}_{piu}\right)$ are velocity of point P_1i_ in the moment of entry to- and exit from ring ku.

Normalizing the velocity and tangent acceleration distributions can be accomplished by following identical steps as those used to normalize the contact density distribution:

10 $${{\text{d}}^{\prime }}_{\text{v}}\left({R}_{D}\right)=\frac{{d}_{\text{v}}({R}_{D})}{{d}_{\text{v}}(R)}$$

11 $${{\text{d}}^{\prime }}_{\text{a}}\left({R}_{D}\right)=\frac{{d}_{\text{a}}({R}_{D})}{{d}_{\text{a}}(R)}$$

The comprehensive evaluations based on the workpiece-tool contact intensity, distribution of its velocity and acceleration, and contact density have been used to determine the wear of tool along the active surface. Based on the results, the intensity of wear can be determined in specific regions of the tool by correlating results of the contact density of the workpiece-tool and considering velocity and tangential acceleration. Calculations were performed to determine the kinematic configurations that result in highest contact intensity along the active surface of the lapping tools. The area with the highest contact density, velocity, and acceleration was used to identify the location with the highest tool wear.

**Fig. 6:**
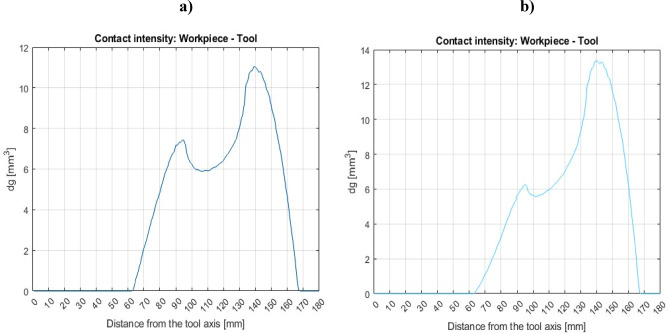
Workpiece-tool contact intensity: (**a**) co-rotational lapping, (**b**) counter-rotational lapping.

**Fig. 7:**
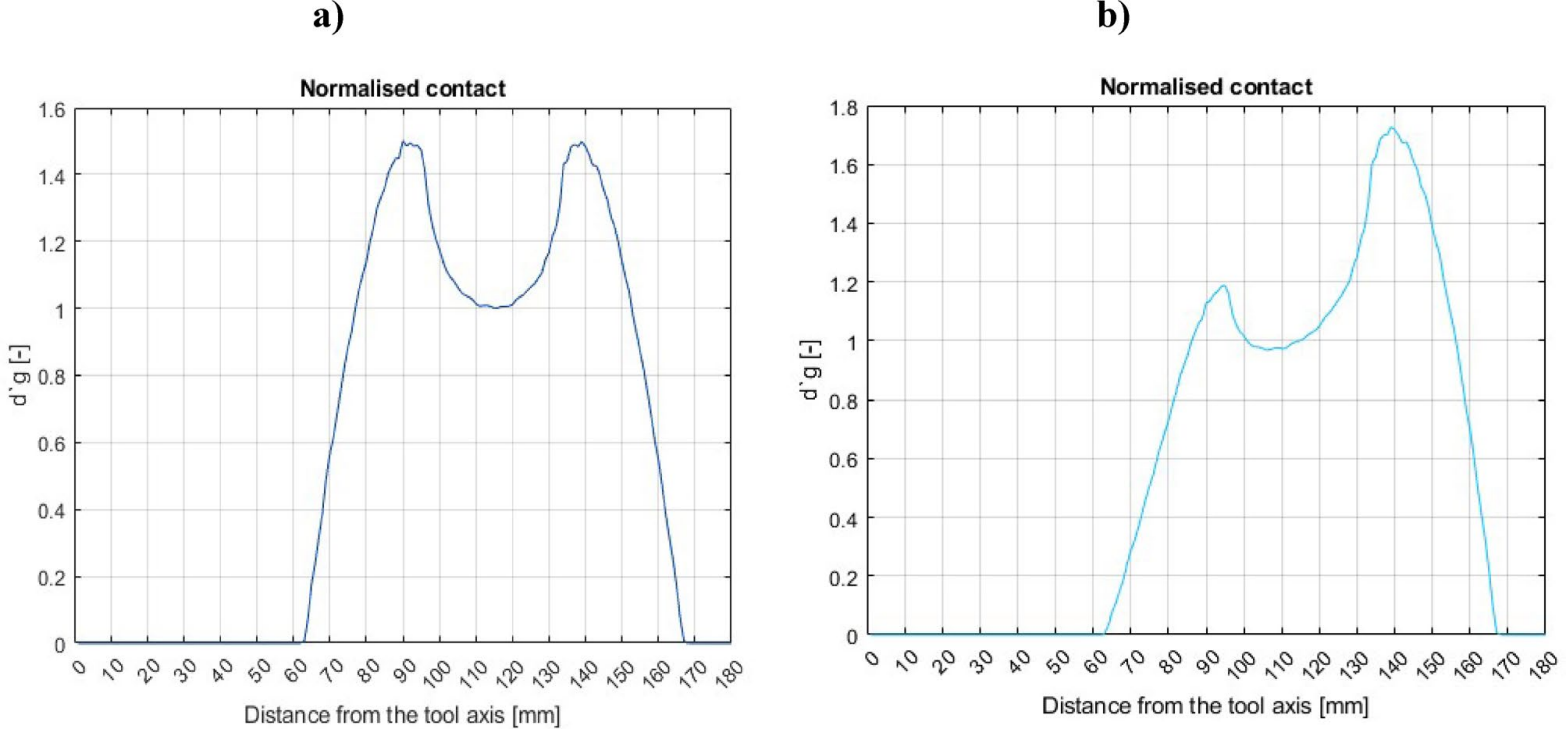
Normalized contact density of (**a**) co-rotational lapping and (**b**) counter-rotational lapping.

**Fig. 8:**
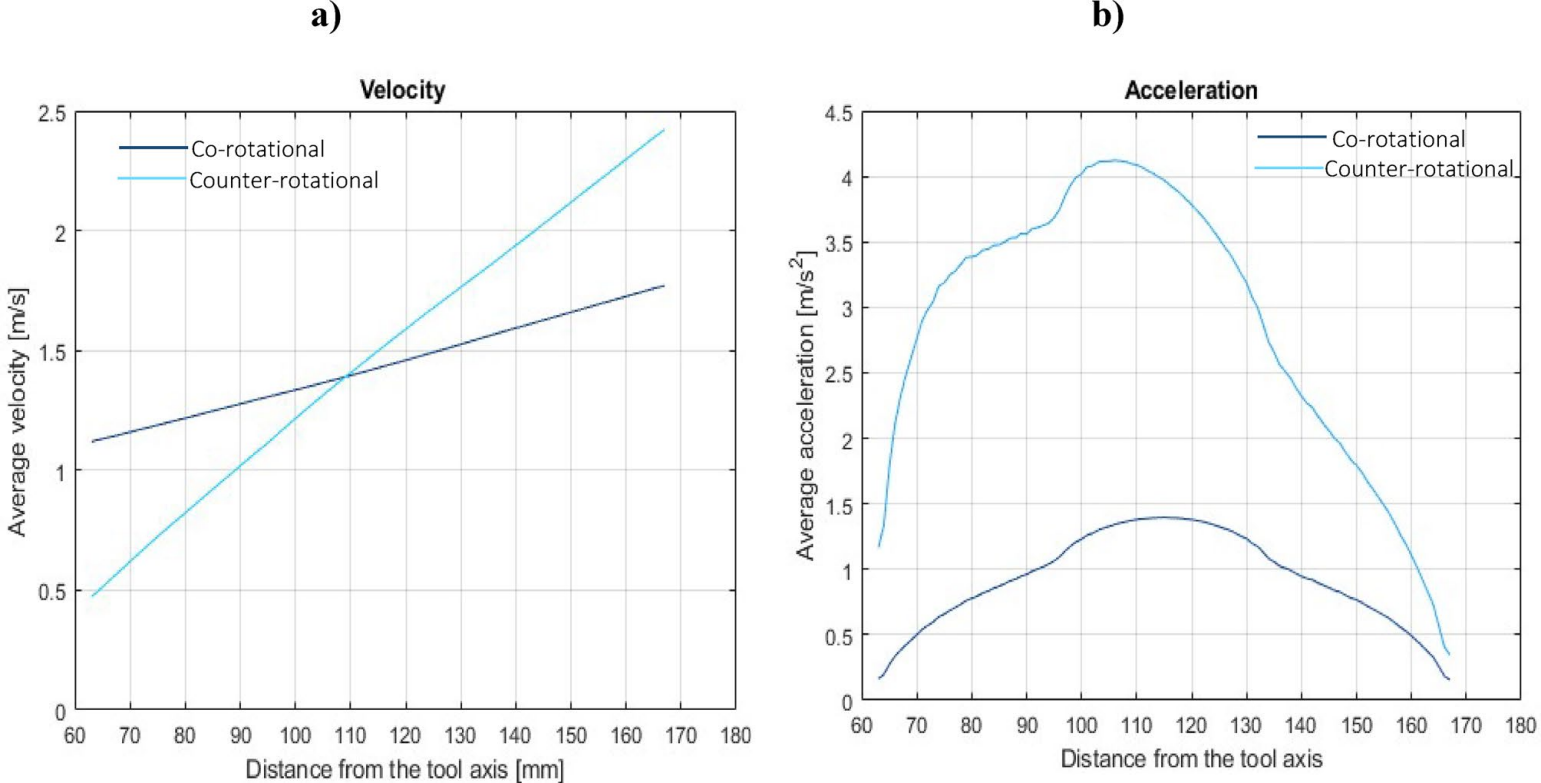
Co- and counter-rotational distribution of (**a**) velocity and (**b**) acceleration.

During the co-rotational lapping, the workpiece-tool contact intensity modeling result (Fig. 6a) shows the highest contact area close to the outer tool radius edge, while the normalized contact density (Fig. 7a) reveals the highest contact density near the inner and outer radius of the tool. The workpiece’s contact intensity and density can be used to establish the location with the maximum workpiece-tool contact area, but this becomes more comprehensive when velocity and acceleration are considered. As indicated in Fig. 8a, during co-rotational lapping, velocity distributions were found to be higher near the outer radius of the tool, but acceleration distributions were found to be higher in the 80–140 mm tool radius range (Fig. 8b). During counter-rotational lapping, the kinematics distribution of contact intensity (Fig. 6b), normalized contact density (Fig. 7b), and velocity distribution (Fig. 8a) was found to be higher near the outer tool radius, whereas the distribution of acceleration was higher in the range of the tool radius, similar to co-rotational 80–140 mm as shown in Fig. 8b, but with a higher intensity. Abrasive particles were found embedded deeper into the active surface of a polyamide tool^26^, and this can be expected due to the resulting highest acceleration forces in the specific area. This embedment of abrasive particles during both kinematics configurations can disrupt the uniform contact between the workpiece and the tool, leading to increased wear. Additionally, acceleration forces can increase the pressures of contact between the lapping tool and the workpiece, resulting in higher abrasive forces and, as a result, intense tool wear in that area. According to the overall calculation results from the modeling, a counter-rotational setup was found to result intense tool wear due to the increased acceleration and contact density along the tool’s active surface. This modeling results proven the experimental tool wear assessments based on minimum zone (MZ) solutions.

## Results and discussion

### Evaluation of tool wear along radial axis of the lapping segments

**Fig. 9:**
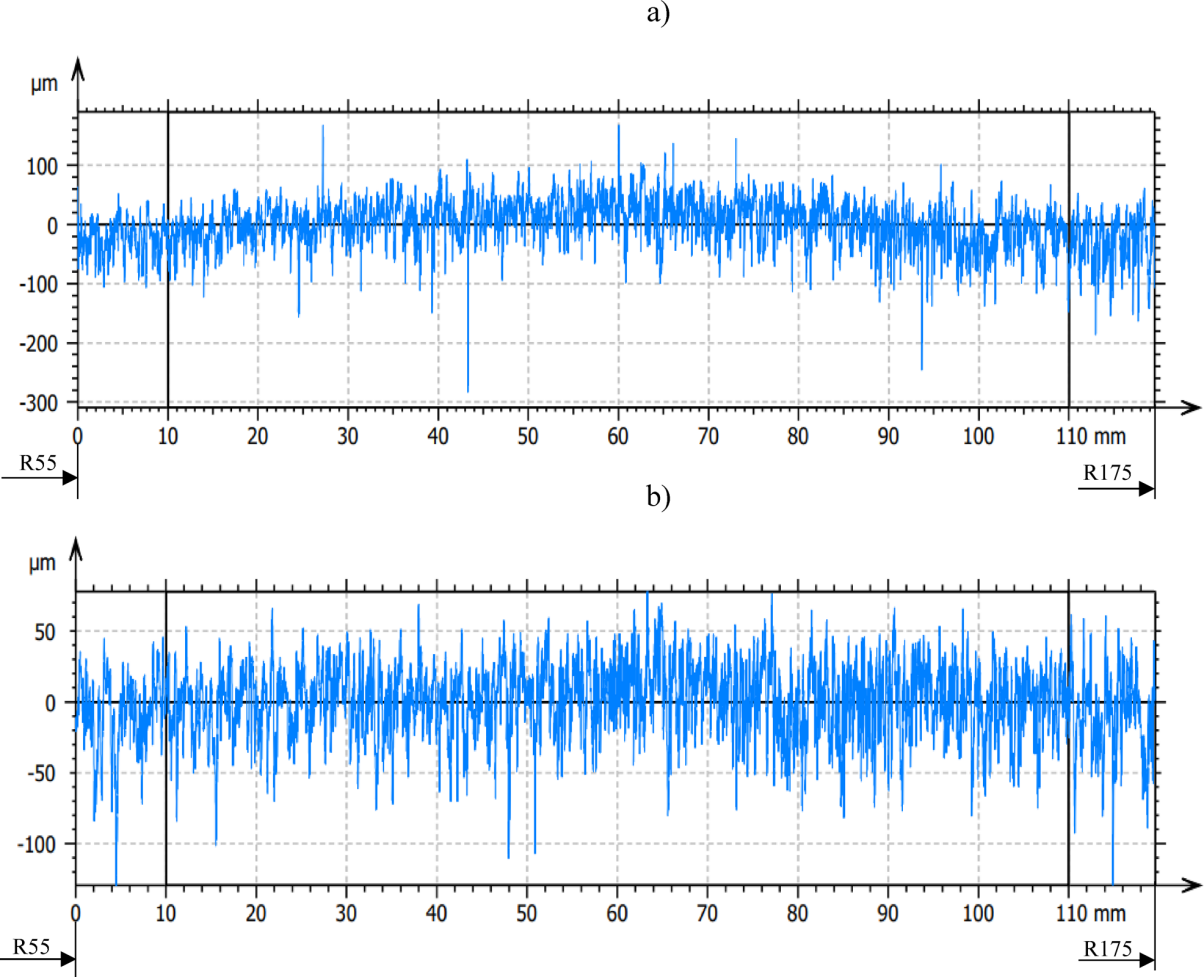
Exemplary profile of the lapping tool segment no. #1 before machining along 120 mm radial length: (**a**) before co-rotational lapping, (**b**) before counter-rotational lapping.

3D-printed polyamide lapping tools composed of eight segments were utilized in the experimental study for each kinematic configuration. Assessment of the segment’s straightness condition after SLS-printing was conducted based on the surface profile according the procedures described in section “Materials and methods”. The measurements are conducted for a total length of 120 mm along radial axis of the segments from the radius of 55–175 mm. Figure 9 illustrates exemplary radial axis profiles of lapping segments #1 before machining for co- (Fig. 9a) and counter-rotational lapping (Fig. 9b). The measured results illustrate that the polyamide lapping segments have a straighter surface profile before machining the Al_2_O_3_ ceramic material samples.

**Fig. 10:**
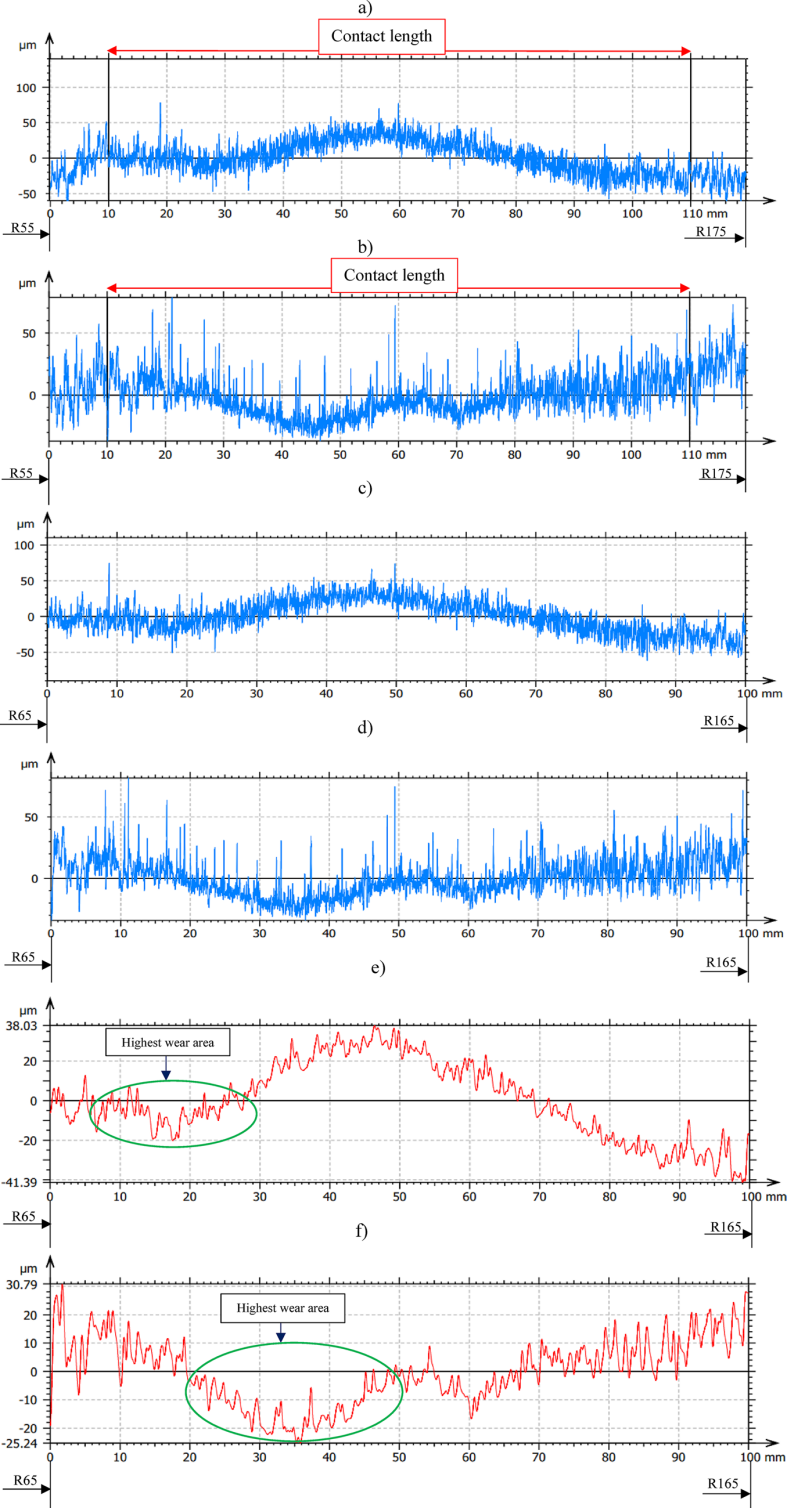
Exemplary raw profile of the lapping tool segment no. #1 after first series (120 min) machining: (**a**) raw profile with 120 mm along radial length– co-rotational lapping, (**b**) raw profile with 120 mm in length– counter-rotational lapping, (**c**) raw profile of the contact area– co-rotational lapping, (**d**) raw profile of the contact area– counter-rotational lapping, (**e**) waviness profile of the contact area– co-rotational lapping, (**f**) waviness profile of the contact area– counter-rotational lapping.

Machining of Al_2_O_3_ ceramic samples and assessment of polyamide lapping tools surface characteristics were conducted for three series with each kinematics configuration; each consecutive series holds 120 min of machining time. Figure 10 shows the lapping tool’s profile after the first series of machining for 120 min with co- and counter-rotational kinematic configurations. As illustrated in Fig. 10a, which is the full-length surface profile along the radial length of segment #1 from the co-rotational lapping of the ceramic materials. To determine the effect of lapping process based on the contact intensity, the segments contact length profile is extracted from the full-length measurement, as indicated in Fig. 10c. The wear characteristics of lapping tools were assessed based on their waviness profile, which is extracted from the raw profile along the contact length. As seen in Fig. 10e, the lapping segments were observed changing straightness from the initial conditions after 120 min of lapping with co-rotational configurations. The result indicates that along the radial length, the range of radius points 70–100 mm has the highest contact intensity, which can be expected as the area for the highest tool wear.

The wear characteristics evaluation of the lapping tool segments was conducted after the first series of machining with counter-rotational kinematics configurations, as shown in Fig. 10b, with a full measured length profile. Following the same procedure used for co-rotational lapping segments profile extraction, the profile of lapping segment #1 after counter-rotational lapping of the ceramic samples was extracted based on the contact length as shown in Fig. 10d. The waviness profile is used to evaluate the wear characteristics of the polyamide lapping segments after the 120 min of lapping the ceramic materials, as shown in Fig. 10f. The kinematics condition resulted in the surface of the segment acquiring different profile while comparing it to the co-rotational configuration, and the highest contact intensity was observed in the range of the radius of the tool, 85–115 mm, which can be expected as the area of the highest tool wear.

**Fig. 11:**
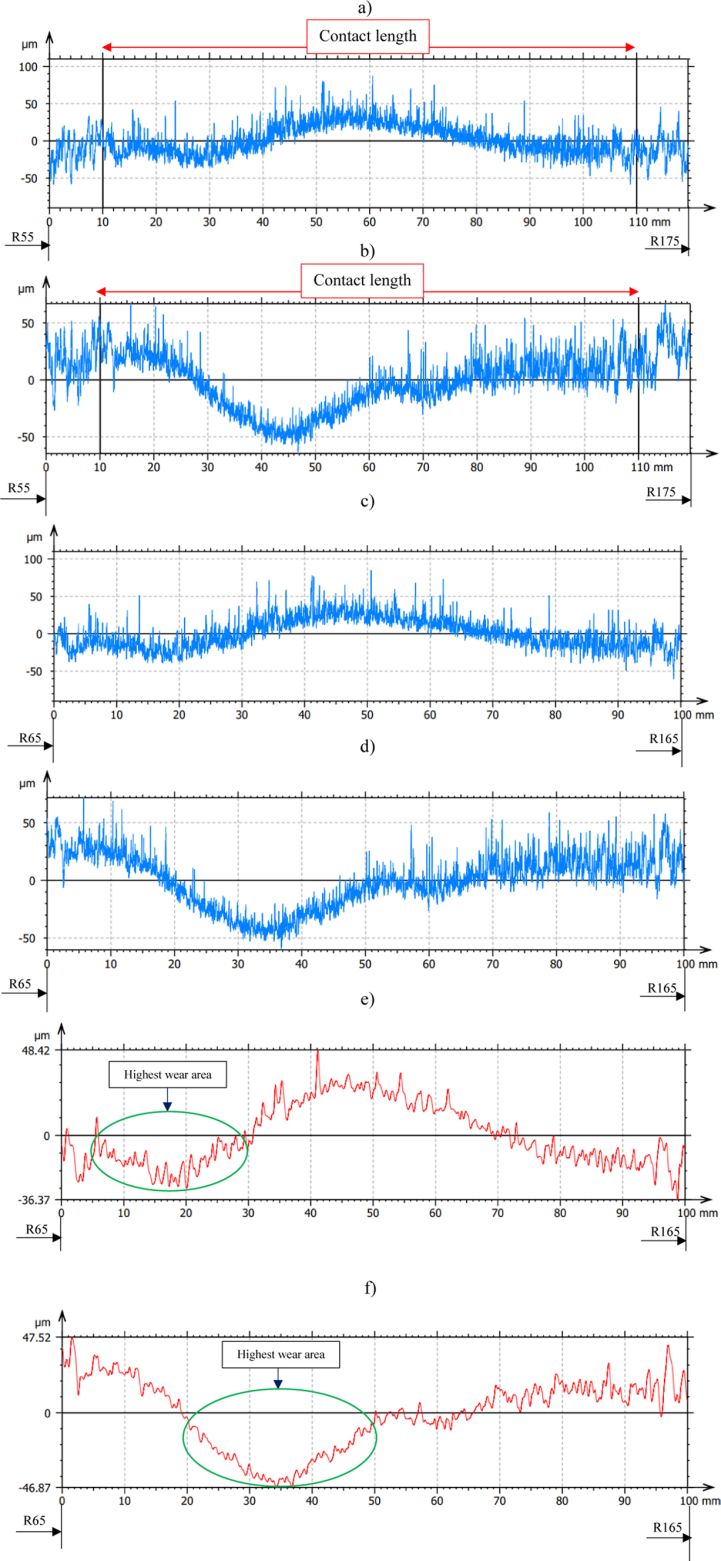
Exemplary raw profile of the lapping tool segment no. #1 after second series (240 min) machining: (**a**) raw profile with 120 mm in length– co-rotational lapping, (**b**) raw profile with 120 mm in length– counter-rotational lapping, (**c**) raw profile of the contact area– co-rotational lapping, (**d**) raw profile of the contact area– counter-rotational lapping, (**e**) waviness profile of the contact area– co-rotational lapping, (**f**) waviness profile of the contact area– counter-rotational lapping.

**Fig. 12:**
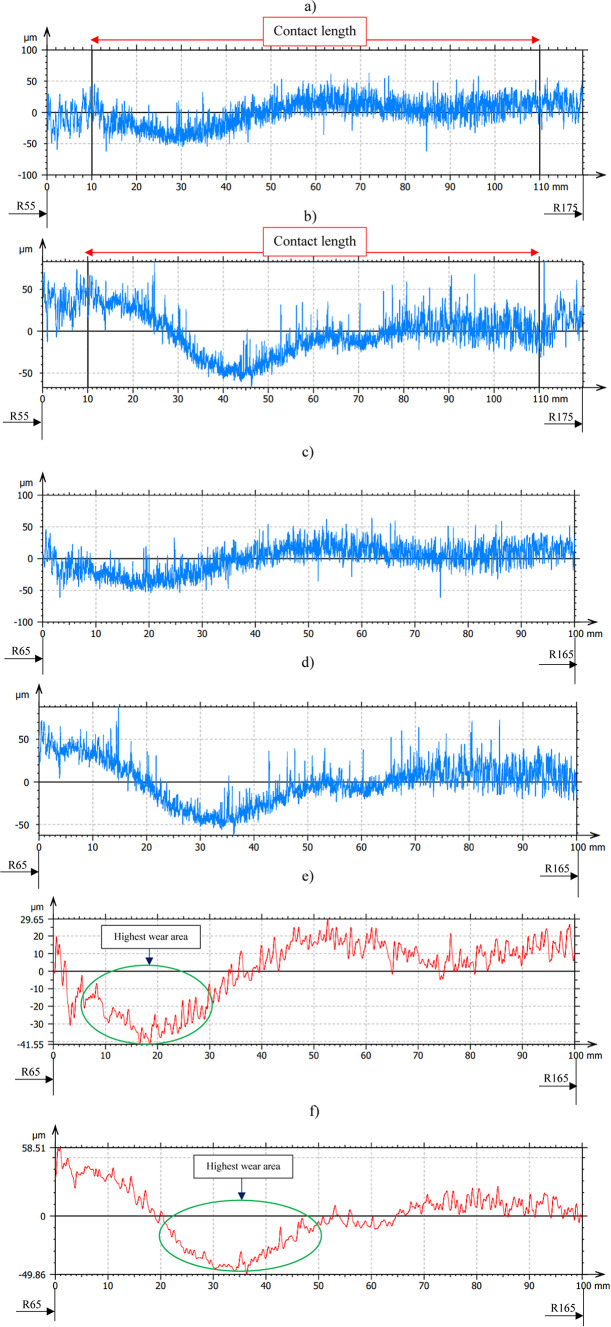
Exemplary profile of the lapping tool segment no. #1 after third series (360 min) machining: (**a**) raw profile with 120 mm in length– co-rotational lapping, (**b**) raw profile with 120 mm in length– counter-rotational lapping, (**c**) raw profile of the contact area– co-rotational lapping, (**d**) raw profile of the contact area– counter-rotational lapping, (**e**) waviness profile of the contact area– co-rotational lapping, (**f**) waviness profile of the contact area– co-rotational lapping.

The influence of three-body abrasion kinematics has been observed on resulting progressive shape error change on the polyamide tool surface even after 240 min of machining—Fig. 11 and after 360 min of machining—Fig. 12 for both co- and counter-rotational configurations. During co-rotational lapping, as seen in Fig. 11e, the shape error profile of segment #1 after 240 min of machining was found to be changing while comparing the profile of shape error after 120 min of machining (Fig. 10e). This shape error of the tool was further influenced by the three-body abrasion, and the measurements after 360 min of machining revealed a significant change in profile shape, resulting in a maximum surface height parameter Wt of 71.2 µm, as shown in Fig. 12e. The counter-rotational kinematics configuration also found in significantly influencing the shape error. The profile in Fig. 11f revealed changes in shape error of the polyamide segment #1 after 240 min of machining while comparing it to the profile after 120 min of machining (Fig. 10f); this change in shape error was observed increasing after 360 min of machining, as shown in Fig. 12f, resulting in a maximum surface height parameter Wt of 108.37 µm. During this progressive change in the shape error of the tools profile for both kinematics configurations, the highest wear location has remained at the same location while increasing in its intensity.

**Fig. 13:**
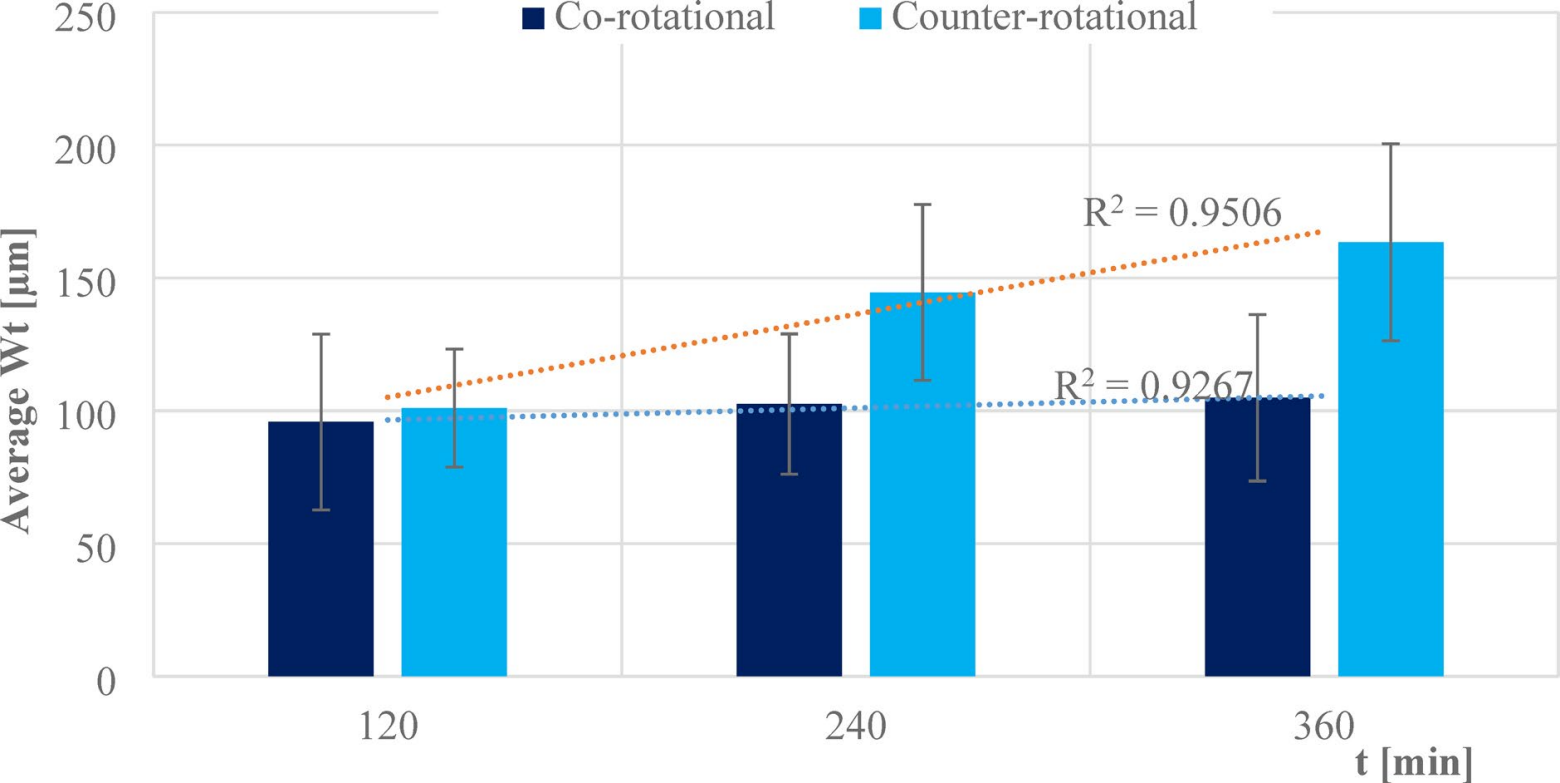
Average maximum height (Wt) along the contact length of polyamide lapping segments after each series of machining with co- and counter-rotational kinematics configurations.

The influence of three-body abrasion kinematics was further assessed for the overall plate condition after consecutive series of machining based on the waviness maximum height Wt parameter and averaging the eight segments measurement for each kinematics configuration. The effect of three-body abrasion during co-rotational lapping was found to influence the shape error of polyamide tools progressively with consecutive machining time, as shown in Fig. 13. This effect was observed, resulting in 95.8 µm of the average maximum height after 120 min, 102.56 µm after 240 min, and 104.89 µm after 360 min of machining. Similarly, during counter-rotational lapping, the surface characteristics of polyamide lapping plate were adversely influenced due to the three-body abrasion while progressively increasing its shape error with machining time. The average maximum height Wt was observed increasing after each series of tests, resulting in 100.98 µm, 144.58 µm, and 163.48 µm after 120, 240, and 360 min of machining, respectively. In each series of experiments, counter-rotational kinematics configuration was found to intensively affect the surface characteristics of the polyamide tool and, as a consequence, result in accelerating the wear on the active surface of the tool.

#### Surface topography of the lapping tools flat surface

Surface topography measurements have been conducted for the lapping segments after the third series of machining, about 360 min of machining the ceramic material Al_2_O_3_ with co- and counter-rotational kinematics configurations. These topography measurements provide valuable insights into the wear characteristics of the lapping segments, enabling the highest contact locations along the radial axis of the tools. The value of measured spatial roughness (Sa) is used to evaluate the contact intensity on the specific radius of the tool following the procedures indicated in section “Techniques used to acquire a radial axis profile and topography of the tool”.

**Fig. 14:**
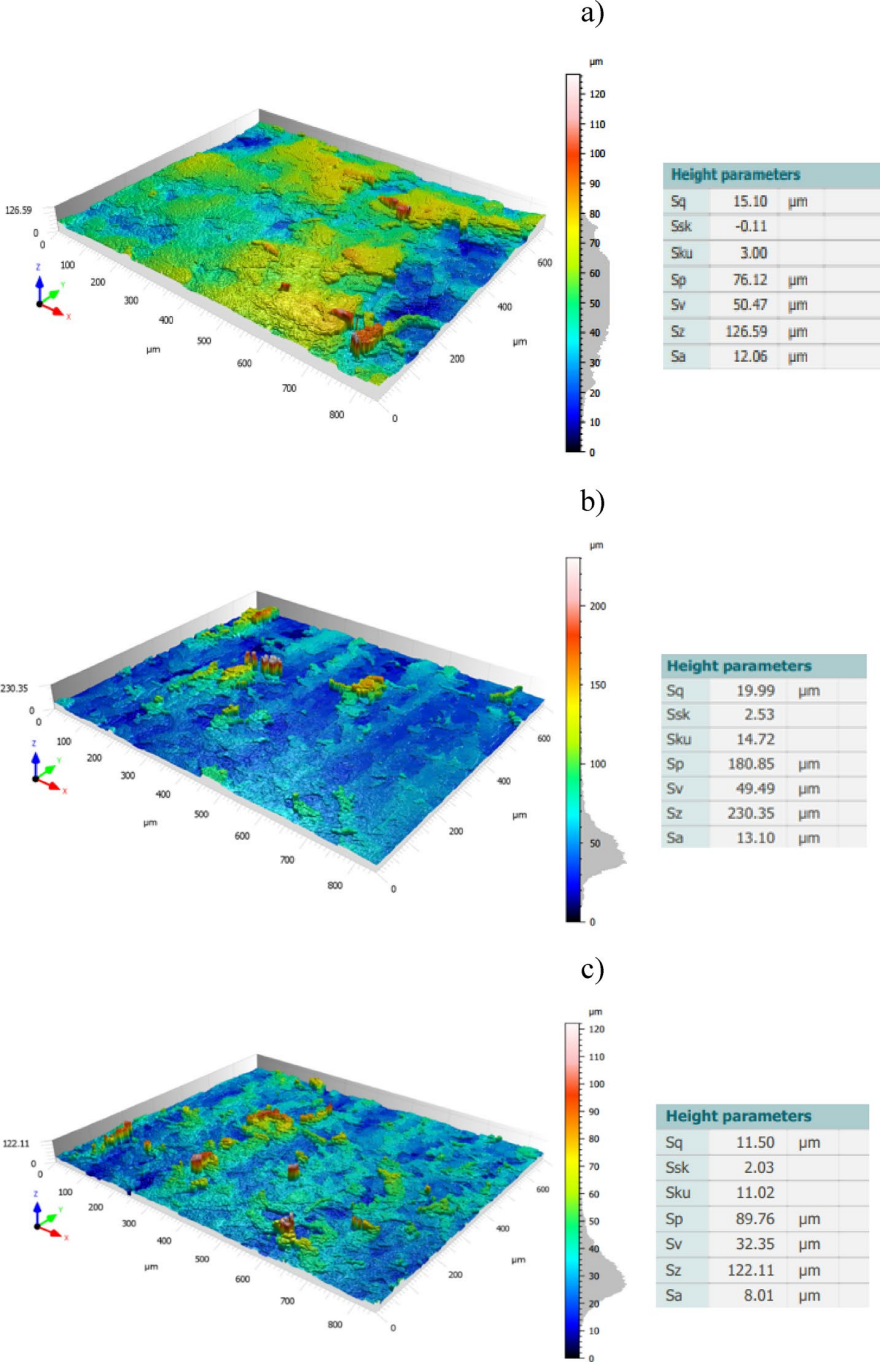
Topography of an exemplary lapping segment #1 after the third series (360 min) of co-rotational lapping in the measuring radial length: (**a**) 185 mm, (**b**) 95 mm, (**c**) 85 mm.

As seen in Fig. 14, the topography of lapping segment after the third series (360 min) of machining with co-rotational kinematics configuration is used to determine the highest contact areas. The measurement results in Fig. 14c shows the surface height parameter at a radius of 85 mm, and it has the lowest spatial roughness with a value of 8.01 µm, indicating a region with the highest contact intensity while comparing it to the other radial points for co-rotational machining, i.e., radius 185 mm, as shown in Fig. 14a, which is out of the contact region with Sa of 12.06 µm, and radius 95 mm in the contact region with Sa of 13.10 µm—Fig. 14b.

**Fig. 15:**
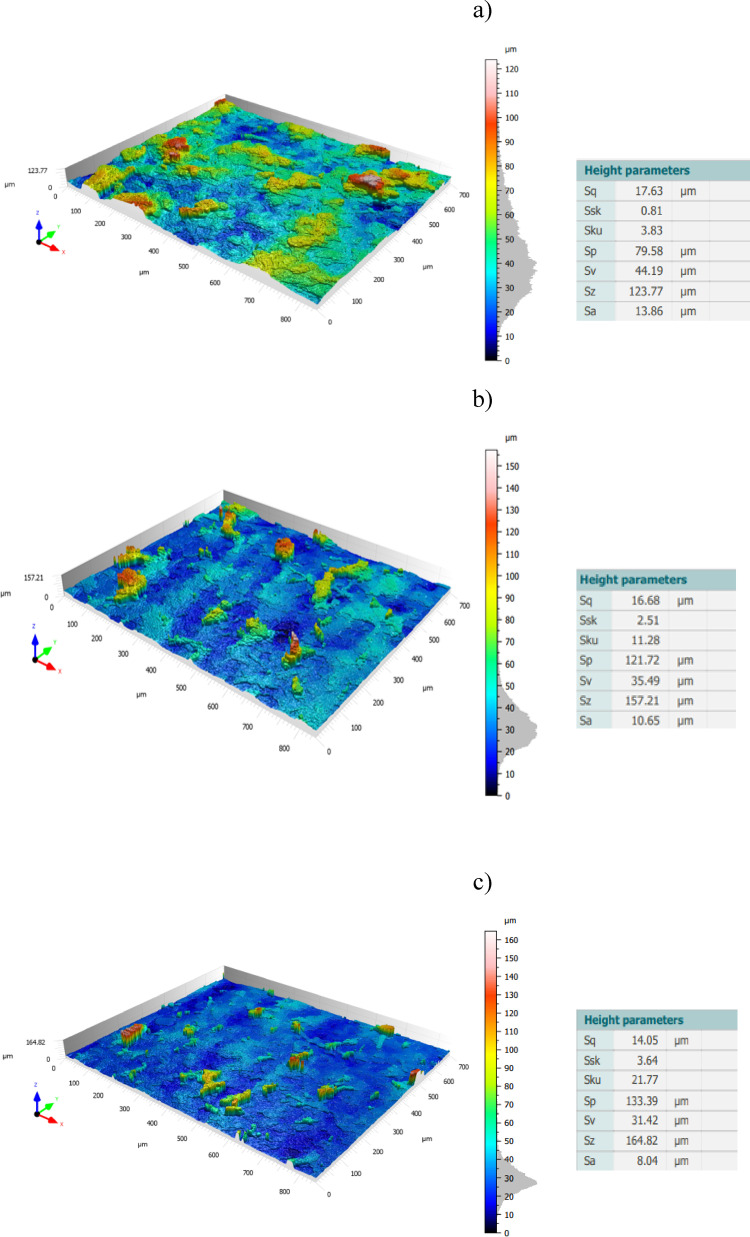
Topography of an exemplary lapping segment #1 after the third series (360 min) of counter-rotational lapping in the measuring radial length: (**a**) 185 mm, (**b**) 85 mm, (**c**) 95 mm.

The surface topography after the third series (360 min) of machining the ceramic materials Al_2_O_3_ with counter-rotational lapping, as shown in Fig. 15, was used to determine the highest contact areas along the radial points of the polyamide lapping segments. The non-contact area of the tool was observed to have the highest spatial roughness Sa value with 13.86 µm at the radius of the tool 185 mm, while the lowest was at radius of the tool 95 mm with the spatial roughness of Sa 8.04 µm, as shown in Fig. 15a,b, respectively. The highest contact area during the co-rotational lapping was found at a radius of 85 mm with spatial roughness Sa of 8.01 µm, whereas during counter-rotational lapping, this area resulted in the lowest contact intensity, having a value of spatial roughness of 10.65 µm, as shown in Fig. 15b. These results revealed the variety of the contact mechanisms of the two configurations, resulting in different highest contact regions on the tool surfaces.

**Fig. 16:**
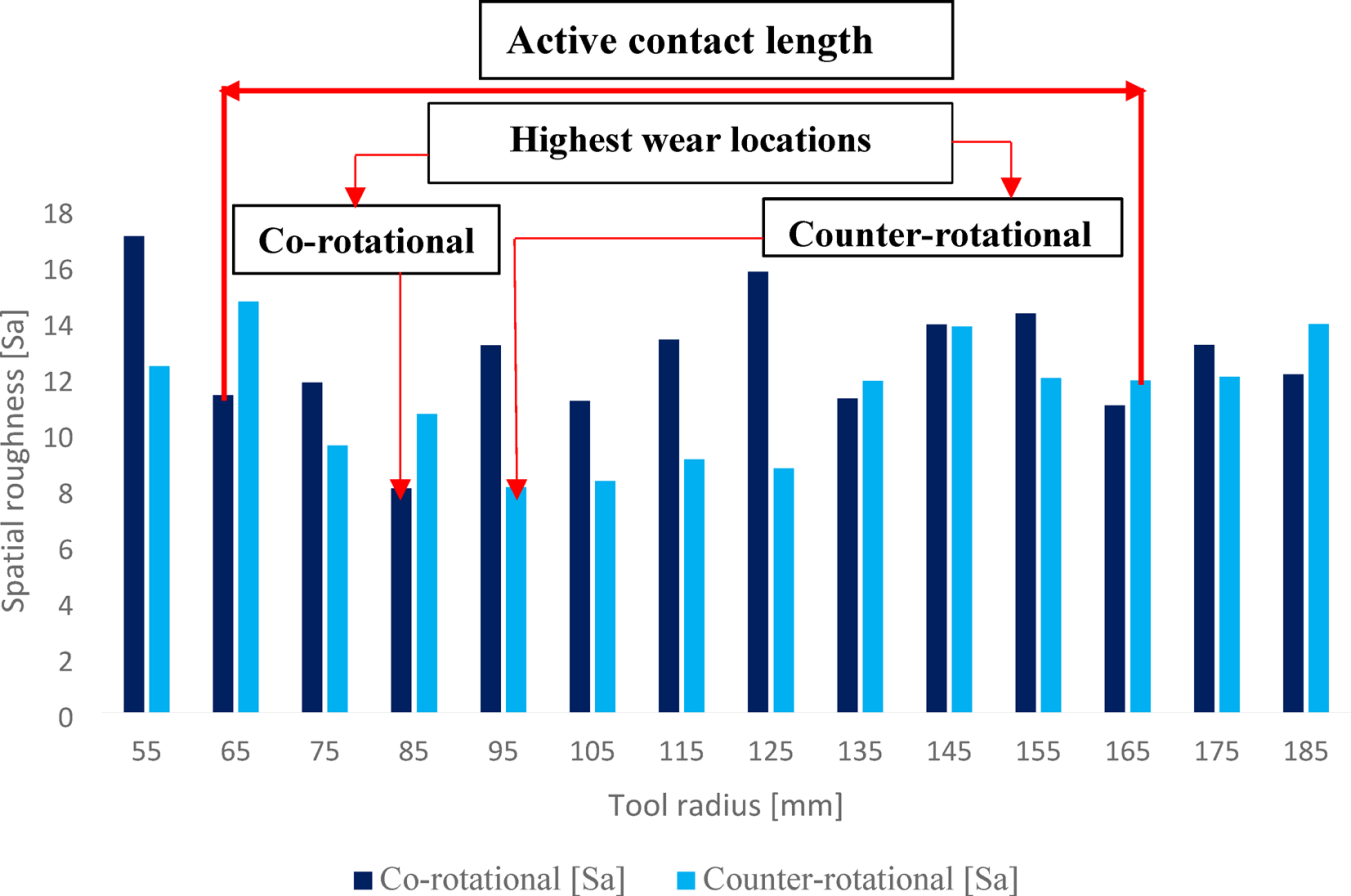
Surface roughness (Sa) of an exemplary lapping segment #1 along the radial points after the third series (360 min) of co- and counter-rotational lapping.

The three-dimensional surface topography results obtained from the polyamide exemplary lapping segment #1 after 360 min of machining ceramic materials with co-rotational and counter-rotational kinematics configuration are used to evaluate the wear characteristics of the tools. The surface height parameter spatial roughness (Sa) from the results of each kinematics configuration with the lapping tool segment #1, as shown in Fig. 16, was used to determine the surface characteristics of the tool. In both kinematics configurations, the surface characteristics have a varied roughness condition as we move from the tool’s inner radius to the outer radius. However, more radial points were observed with the lowest roughness (Sa) values during counter-rotational kinematics. This evidence confirms that during counter-rotational lapping, the tool had wide and high contact areas with the workpiece due to its different kinematics contact mechanism. Table 5 gives the roughness values for the selected radial points of the segments. The non-contact areas of the tools were identified as radii of 55, 175, and 185 mm, with the highest roughness values for both kinematics configurations, and the radii of 85 mm and 95 mm of lapping segments were observed to have the highest contact intensity during the co-rotational lapping and counter-rotational lapping, with spatial roughness Sa of 8.01 µm and 8.04 µm, respectively.

**Table 5:** Selected radial point surface roughness parameter Sa of an exemplary SLS-fabricated lapping segment #1.

Tool radius (mm)	Surface roughness Sa for kinematics configuration	
	Co-rotational (µm)	Counter-rotational (µm)
55	17	12.35
85	8.01	10.65
95	13.1	8.04
175	13.11	11.98
185	12.06	13.86

### Technological effects

#### Surface characteristics of Al_2_O_3_ ceramic materials

**Fig. 17:**
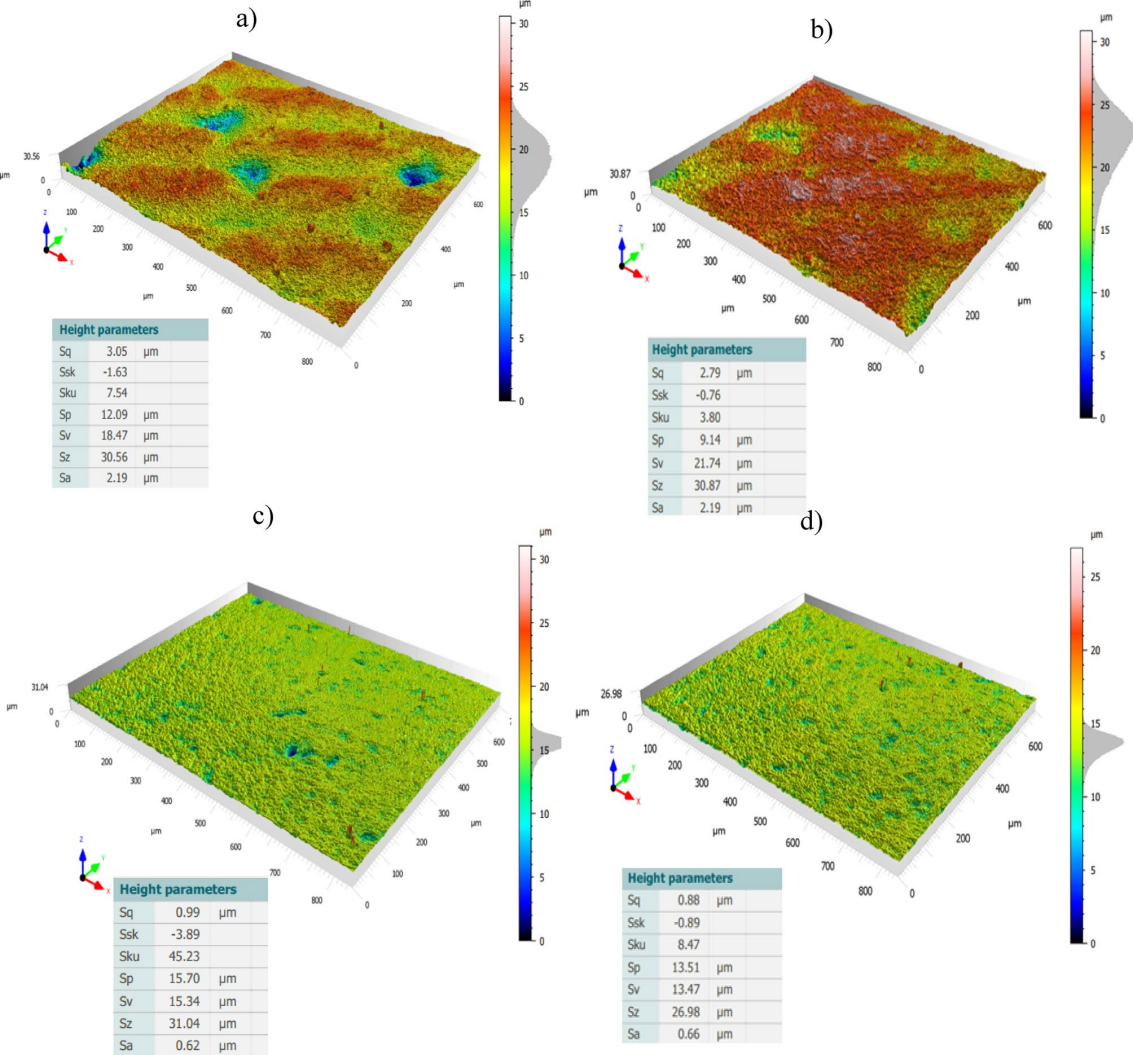
Exemplary three-dimensional surface topography of ceramic materials with their height parameters: (**a**) before co-rotational lapping, (**b**) before counter-rotational lapping, (**c**) after series three (360 min) of co-rotational lapping, (**d**) after series three (360 min) of counter-rotational lapping.

Ceramic materials are categorized under materials of having a porous structure and difficulty of cutting during machining. The adopted set of lapping parameters was utilized for co- and counter-rotational lapping, resulted in enhancing the surface characteristics of Al_2_O_3_ ceramic materials. The measurement results of three-dimensional surface roughness (Sa) of ceramic materials, as shown in Fig. 17, observed decreasing after machining from the initial spatial roughness. As seen in Fig. 17a,c, the spatial roughness of the ceramic sample was decreased after co-rotational lapping of 360 min from the initial condition of roughness Sa 2.19 µm and Sq 3.05 µm to Sa 0.62 µm and Sq 0.99 µm, with 71.7% improvement of the surface roughness. The surface roughness after 360 min of counter-rotational lapping of ceramic materials was also observed to improve from the initial condition of roughness Sa 2.19 µm and Sq 2.79 µm to Sa 0.66 µm and Sq 0.88 µm, as shown in Fig. 17b,d, indicating the surface of the ceramic materials improved by 69.9%.

**Fig. 18:**
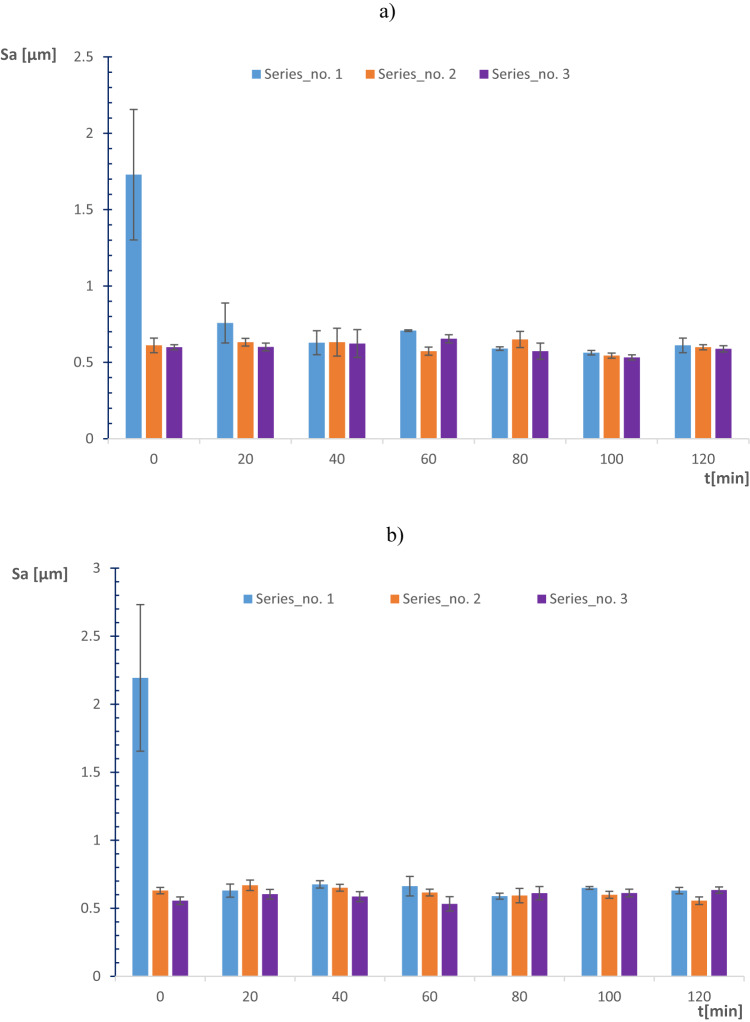
Average spatial roughness (Sa) of Al_2_O_3_ ceramic materials for each series of tests: (**a**) co-rotational lapping, (**b**) counter-rotational lapping.

The surface characteristics of Al_2_O_3_ ceramic materials based on areal surface roughness were evaluated during each series of experiments after a consecutive 20 min of machining. The average sample surface roughness Sa was considered to evaluate the effect of the adopted set of parameters and the performance of 3D-printed lapping segments within co- and counter-rotational kinematic configurations. Ceramic materials roughness was observed to decrease within 20 min of machining during both settings of kinematics configurations, as shown in Fig. 18a,b. The Al_2_O_3_ ceramic materials average surface roughness during the first series (120 min) of machining with co-rotational kinematics configuration observed improving the surface characteristics by 64.74% while decreasing Sa from 1.73 to 0.61 µm—Fig. 18a. However, the surface roughness of the ceramic samples was observed with minor improvement during the second and third series of machining by resulting 2% and 1.67% roughness reduction, respectively.

The results obtained during counter-rotational lapping, as shown in Fig. 18b, emphasize fluctuations of surface roughness improvement during various machining times. The first series of lapping with counter-rotational kinematics configuration resulted in a 71.3% improvement of the average surface roughness, decreasing from the initial Sa of 2.193 µm to 0.63 µm. The second series of machining had resulted in 11.8% of surface enhancement by improving the surface roughness, whereas the third series of counter-rotational kinematics was found to fluctuate the surface roughness after each consecutive 20 min of lapping, resulting in slight deteriorations of surface characteristics. Generally, comparable surface enhancement was obtained with both co- and counter-rotational kinematics configurations on the ceramic material Al_2_O_3_, with continuous improvement of the surface occurring in the three series of co-rotational kinematics configuration and slight fluctuation occurring in the third series of counter-rotational kinematics configuration.

**Fig. 19:**
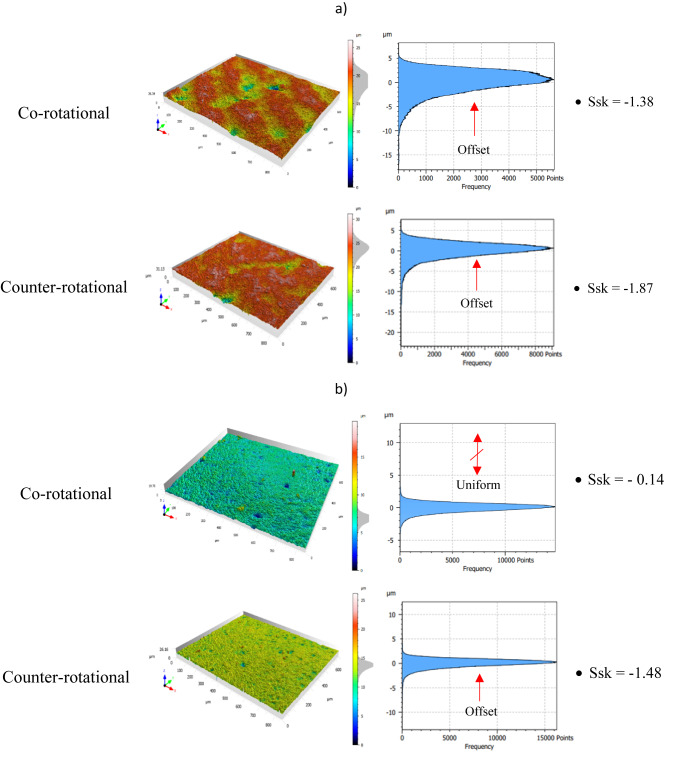

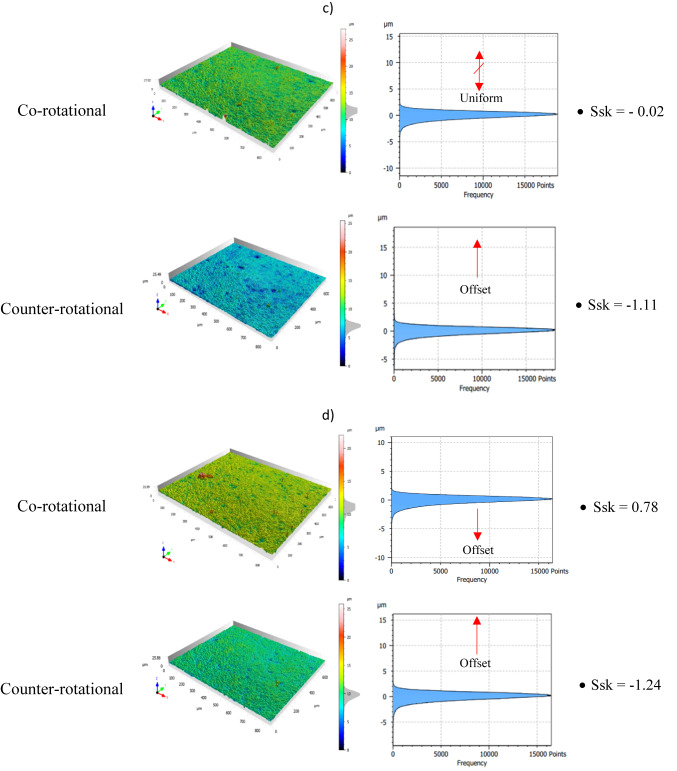
Exemplary height distribution on the surface of ceramic material sample #1: (**a**) before machining, (**b**) after series no.1 (120 min), (**c**) after series no. 2 (240 min), (**d**) after series no. 3 (360 min).

Evaluation of the ceramic materials surface characteristics was further conducted based on the surface height distribution. This can help to control the lapping process while ensuring quality scores and selecting appropriate lapping kinematics to achieve the required surface finish. The height distribution was evaluated in the form of histogram charts and a skewness Ssk surface parameter while determining the degree of asymmetry based on the distribution illustration of the histogram chart. The observed surface conditions of the ceramic materials before machining revealed their heights were clustered closer and found above the mean plane of the measured surface. This is confirmed with the results obtained of negative surface skewness Ssk with the value of − 1.38 as shown in Fig. 19a of sample no. 1 before co-rotational lapping. The sample, which is used for counter-rotational lapping, also found in negative skewness Ssk with the value of − 1.87, revealing the surface heights clustered closer and found above the mean plane. This negative Ssk determines that ceramic material surfaces before machining have fewer valleys but deeper, and most of the peaks are clustered closer or found above the mean plane of the surface.

The surface height distributions on the ceramic samples were examined after each series of 120 min of lapping during both kinematics configurations, and the findings revealed the effect of kinematics configuration on obtaining uniform surface height distributions. As seen in Fig. 19b, the surface skewness after series one (120 min) of machining with co-rotational kinematics configuration found near to zero with the value of Ssk − 0.14, which implies the height are uniformly or symmetrically distributed around the mean plane of the surface. Whereas the height distribution after 120 min of lapping with counter-rotational kinematics configuration found negative skewness with the value of − 1.48, revealing a slight improvement from the initial condition. The second series of machining resulted in the same effect as the first, by resulting symmetrical surface height distributions during co-rotational kinematics configuration, whereas counter-rotational found with negative skewness Ssk of − 1.11 having more peaks distributed above the mean plane and with higher values of fewer valleys. During the second series of co-rotational lapping, the skewness was found to be − 0.02 but much closer to zero—Fig. 19c, and this implies the surfaces height are uniformly distributed around the mean plane. The results obtained after the third series of machining the ceramic sample indicated the surface height distribution after 360 min of machining with counter-rotational lapping was nom-uniform around the mean plane, whereas co-rotational kinematics configuration resulted in positive skewness with the value of Ssk 0.78, which implies the surface was distributed slightly below the mean plane and fewer peaks skewed above the mean plane of the surface, as shown in Fig. 19d. Overall, the ceramic materials surface height distributions were improved significantly with co-rotational kinematics configurations by resulting in near-zero surface skewness.

#### Evaluation of material removal from the surface of Al_2_O_3_ ceramic materials

Material removal during the lapping process mainly occurs due to the mechanical abrasion between the ceramic materials and polyamide lapping plate with introduced loose diamond abrasive particles, as discussed in section “Material removal mechanism of single-sided free abrasive flat lapping”. The removal of material can be influenced by the abrasives size and shape, lapping plate property, load and pressure, speed of lapping, and surface conditions or flatness of the polyamide lapping plate.

**Fig. 20:**
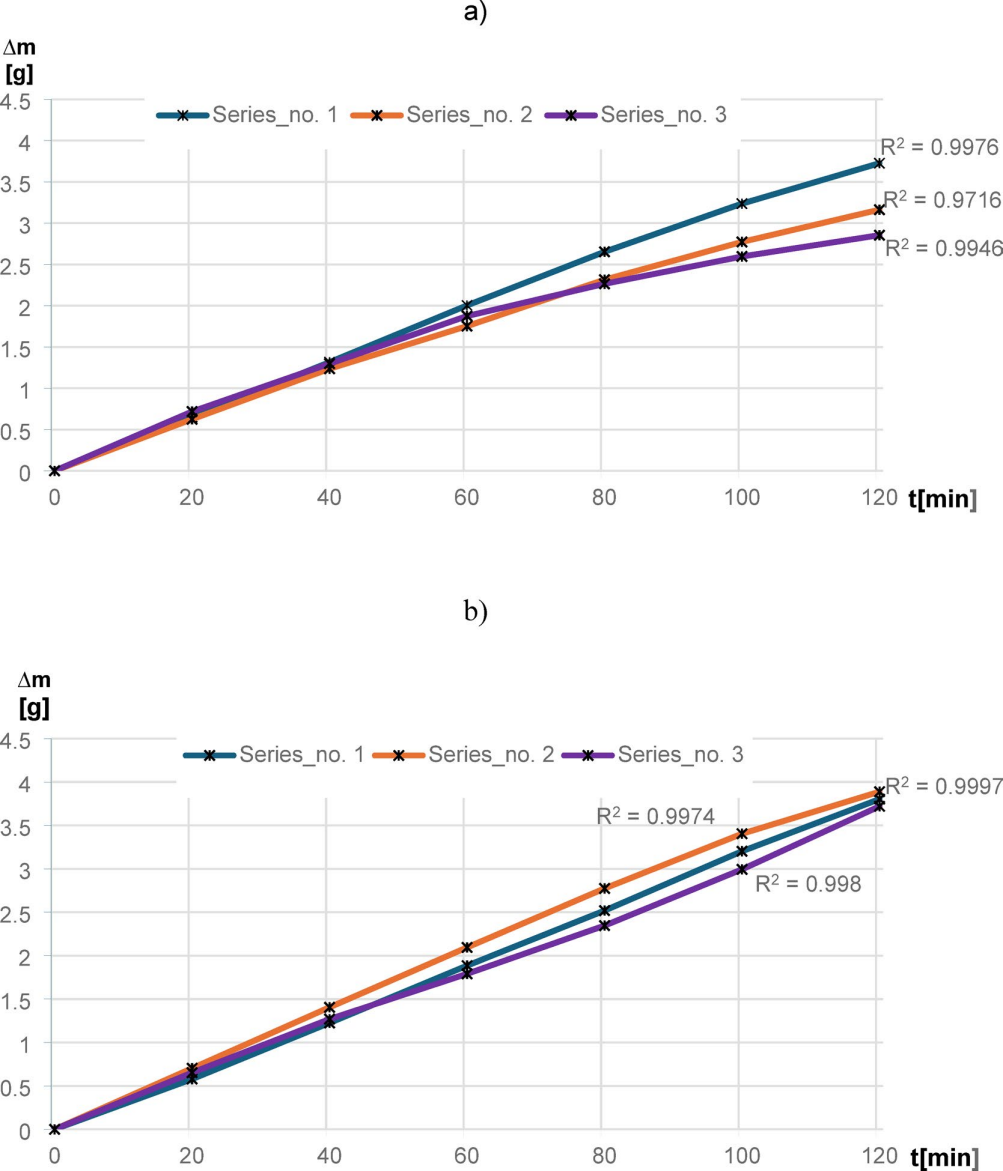
Mass material removal of the ceramic material Al_2_O_3_ for each series of tests: (**a**) co-rotational lapping, (**b**) counter-rotational lapping.

The removal of materials was measured after each consecutive 20 min of machining time using a digital balance, and the charts are drawn for each kinematic configurations as shown in Fig. 20. Co-rotational kinematics configuration resulted in a significant removal of materials with linear incremental form during each series of machining—Fig. 20a. A total of 9.74 g of material was removed from the surface of Al_2_O_3_ ceramic material with kinematics of co-rotational configuration. During the first series of machining, 3.73 g of material was removed from the surface of the ceramic samples, and the tendency of material removal was found to be slightly decreasing after 80 min of machining. The second and third series of machining resulted in lower removal of material, with the values of 3.16 g and 2.85 g, respectively, compared to the first series.

As seen in Fig. 20b, the removal of material obtained with counter-rotational kinematics configuration has a linearity trend through the three series of machining. The highest material removal was observed during the second series of machining, with a value of 3.89 g, while the first series removed an amount of 3.80 g. During the third series, the material removal was observed decreasing by removing 3.72 g of material. However, the total amount of material removed from the ceramic samples with counter-rotational kinematics configuration was recorded as 11.41 g, implying 17.14% higher removal of material when compared to the total material removed during lapping of ceramic materials with co-rotational kinematics configuration.

### Discussion

The influence of three-body abrasion of kinematics on the surface characteristics of SLS-printed polyamide lapping tools and the resulting technological effects were assessed by machining difficult-to-machine ceramic materials, Al_2_O_3_. Following the 3D printing and basic post-processing, like sandblasting operations to remove the unsintered powders from the print, the polyamide lapping segments are directly used by applying the abrasive suspension containing loose diamond D107 grains into the active surface of the plate. In order to allow the wear assessments, abrasive suspensions were cleaned from the tool’s active surface after each series of experiments. The experimental evaluations have been made, divided into a total of three series, each with 120 min of machining for both co- and counter-rotational kinematics configurations, and the results are confirmed by simulations from Matlab®. The kinematics of three-body abrasion was observed influencing the lapping tool made using SLS printing from polyamide powders by resulting in significant wear on the tool’s active surface, and on the other hand, in obtaining enhanced technological effects on the workpiece material Al_2_O_3_.

The assessment of tool wear was conducted continuously measuring along the radial axis of lapping segments while considering the non-contact and contact regions of the tool surface during the machining process. The tool profile is then used to estimate the wear condition of the tool, and this has been obtained using the minimum zone (MZ) method. The extraction of the waviness profile from the raw one was performed using a Gaussian filter with a cut-off 0.80 mm, and the surface height parameter maximum heights Wt is used to determine the wear characteristics of the tool. To account for the initial conditions of the lapping segments after SLS printing, measurements were performed on the segments as shown in Fig. 9. The measurement results proved the tool condition was uniform and rough with its surface property of polyamide materials. Assessments have been conducted on the extracted tool profile, only considering the active contact length of the tools. As seen in Figs. 10, 11 and 12, the tool profile was observed changing with three consecutive series of machining, and the tool profile has indicated the significant tool wear area along the radial length of the segments in both kinematic configurations. These significant changes in tool profile have occurred due to the effect of abrasion of three bodies (workpiece, tool, and diamond abrasive grains) and, as described in^44^, the resulting unevenly highest contact density and acceleration along the tool’s active surface. As seen in Fig. 13, the wear of polyamide tools have been observed progressively increasing with machining time for both kinematic configurations, but the amount of wear was found to be higher during the counter-rotational lapping process, resulting in a higher average maximum height of (Wt) 163.48 µm on the tool surface after the third series of tests (360 min). The assessment of tool wear characteristics in both kinematic configurations further conducted based on the surface topography of the abrasive segments along the radial points, and the results were revealed by pinpointing the highest contact points with the lowest spatial roughness Sa of 8.01 µm at radial point 85 mm for the co-rotational kinematics configuration and radial point 95 mm with Sa of 8.04 µm for the counter-rotational kinematics configuration. These results were also confirmed by the modeling, resulting in the ranges of radial length with highest tangential acceleration, as described in section “Modeling of tool-workpiece contact within kinematics configurations”.

The effect of three-body abrasion was assessed in the resulting technological effects, such as material removal rate and surface characteristics of the ceramic material Al_2_O_3_. Both co- and counter-rotational kinematics configurations have been found to enhance the lapping process, but when it comes to the comparison, the amount of overall material removal after 360 min of lapping was found to be higher by 17.14% during counter-rotational kinematics, significantly improving the removal process. However, the surface roughness was found to be better during the co-rotational lapping process, resulting in lower surface roughness after the third series of tests (360 min) with the spatial roughness Sa of 0.61 µm. Additionally, ceramic sample roughness was observed deteriorating during the third series of machining with counter-rotational kinematics configurations. The surface characteristics of ceramic materials were further investigated by considering the surface height distribution based on the skewness parameter (Ssk) and histogram chart. The results revealed that three-body abrasion kinematics with co-rotational configuration was capable in obtaining uniform surface height distribution while resulting in the skewness Ssk near-zero after 120 min of machining, as indicated in Fig. 19b.

The lapping tools employed in this experimental assessment have been found significant in obtaining the required technological effects as of the conventional lapping process, confirming the results in^22^. Additive manufacturing offered custom design fabrication of tools for various application areas, and this was seen in related studies as discussed in section “Introduction” of the presented paper. This AM-based tool fabrication has been found to be prominent direction for the ongoing advancement of precision industries with abrasive machining. Overall, the influence of three-body abrasion on the surface characteristics of the SLS-printed polyamide lapping tool was assessedwhile machining difficult-to-cut material, i.e., Al_2_O_3_, and the obtained results proved the modeling conducted for tool-workpiece contact within the kinematics configuration.

## Conclusions

The influence of three-body abrasion kinematics has been thoroughly examined on the surface characteristics of additively fabricated polyamide lapping tools during machining of difficult-to-cut Al_2_O_3_ ceramic materials. The kinematics configurations of co- and counter-rotational arrangements were utilized with a set of parameters to assess the surface condition of polyamide lapping tools and the resulting technological outcomes. Considering series of machining with each kinematics configuration and following the analysis of experimental and simulation modeling, the following conclusions are drawn:

1. Considerable influence of kinematic configurations was observed on the surface characteristics of SLS-printed polyamide tools and resulted technological outcomes; however, the tool was found in relatively lower wear for machining a difficult-to-machine material, Al_2_O_3_.
2. The wear rate was seen to be increasing progressively with machining time for both kinematics configurations. The results from the waviness profile based on the minimum zone (MZ) technique after the third series (360 min) of machining revealed the highest tool wear locations on the range of tool radius 70–100 mm during co-rotational kinematics and tool radius 85–115 mm during counter-rotational kinematics. Simulation results revealed that for both kinematic configurations, the highest wear in the specified ranges of the tool radius mainly occur due to the highest contact density and acceleration, causing the wear rate to intensify. These results were further confirmed by the surface topography of tools active surface, revealing radial point 85 mm had a pick wear location with co-rotational kinematics resulting with lowest spatial roughness Sa = 8.01 µm and radial point 95 mm resulting with the lowest spatial roughness of Sa = 8.04 µm with counter-rotational kinematics.
3. The three-body abrasion with co-rotational kinematics has been found to have a relatively lower wear value resulting in the average maximum height value of the waviness parameter Wt = 104.89 μm after the third series (360 min) of machining. Whereas the three-body abrasion during counter-rotational kinematics resulted in an average Wt of 163.48 µm after the third series (360 min) of machining. This higher average maximum height (Wt) with counter-rotational kinematics occurred because of the resulting non-uniform contact density along the radial axis and higher tangential velocity and acceleration, increasing the extent of tool wear rate.
4. Kinematics of three-body abrasion with a set of process parameters has been found to significantly improve the surface characteristics of ceramic materials in both configurations. However, co-rotational kinematics was observed with continuous improvement through the three-series assessments, while counter-rotational deteriorated the surface during the third series (360 min) of machining. The improvement of surface quality that occurred during the three-series of machining with co-rotational kinematics is because of the resulting lower relative velocity, which causes the abrasive to slide or roll consistently between the polyamide lapping tool and Al₂O₃ ceramics, leading to smoother machining and better surface finish.
5. The amount of material removal during the total three series of tests (360 min) of machining was found to be higher with counter-rotational kinematics, resulting in 17.14% more material removal from the surface of the ceramic. The resulting higher relative velocity during counter-rotational lapping was expected to lead to the occurrence of better material removal. Since higher relative velocity results in faster three-body abrasion action, causing more intensive embedment of the abrasive grains on the flat surface of the polyamide tool and enhancing the material removal.
6. The surface height distributions were further analyzed based on histogram and surface skewness parameter Ssk. The co-rotational kinematics was found to significantly enhance the ceramic material’s uniform surface distribution by resulting in a surface skewness Ssk near-zero after 120 min of machining.

Overall, the influences of three-body abrasion kinematics on the surface characteristics of SLS-printed lapping tools were examined experimentally, and simulation was performed to model the tool-workpiece contact considering the workpiece velocity and tangential acceleration. Machining of ceramic materials Al_2_O_3_ with co-rotational kinematics was found in lower wear on the tools active surface while improving the surface characteristics of ceramics. In contrast, the counter-rotational kinematics resulted in intense wear on the tool’s active surface while enhancing the material removal process. The workpiece-tool contact modeling confirmed the experimental observations by considering the influence of velocity and tangential acceleration of the workpiece trajectory distribution along the tool’s active surface. Further evaluations of various kinematic parameters with long-lasting machining time and different kinematic configurations will give insight into the ongoing advances of tool fabrication using additive manufacturing, accounting for the uniformity of tool wear. Additionally, optimizing the printing parameters will enhance the operational efficiency of the polyamide lapping tool while improving the wear resistance.

## Data Availability

The data that support the findings of this study are available from the corresponding author, [SWA], upon reasonable request.
